# Body-composition phenotype axes provide additional explanatory information for cardiorespiratory fitness and liver-related metabolic risk within BMI strata

**DOI:** 10.3389/fnut.2026.1881228

**Published:** 2026-08-31

**Authors:** Yang Zheng, Ying-Yi Chen, Hao-Yuan Xu, Rui-Jia Li, Qian You, Xiao-Fan Jing, Feng-Mei Yu, Zhi-Yong Rao

**Affiliations:** Department of Clinical Nutrition, West China Hospital, Sichuan University, Chengdu, China

**Keywords:** alanine aminotransferase, BMI, body composition, body-composition phenotyping, estimated VO_2_max, phase angle

## Abstract

**Background:**

Body mass index (BMI) does not fully characterize heterogeneity in adiposity patterning, lean reserve, and impedance-derived body-composition features. We derived residualized body-composition phenotype axes from routine measurements and examined whether these projected axes provided additional explanatory information for estimated VO₂max and alanine aminotransferase (ALT) within BMI strata.

**Methods:**

Phenotype axes were derived using principal component analysis (PCA) of six variables (BMI, waist-to-height ratio, fat mass index, fat-free mass index, percent body fat, and phase angle) following residualization for age and sex in 23,698 individuals. Robustness and repeatability were assessed. The derivation-defined axes were externally projected into 5,060 adults aged 20–49 years from the National Health and Nutrition Examination Survey. Survey-weighted regression models were used to examine associations between projected phenotype scores and estimated VO₂max from the submaximal treadmill fitness examination (*n* = 2,721) and alanine aminotransferase (ALT) (*n* = 4,857) within unified BMI quartile strata, with adjustment for age, sex, and race/ethnicity. Sensitivity analyses included expanded covariate adjustment, false-discovery-rate correction, interaction testing, protocol-adjusted estimated VO₂max models, and ALT clean-sample analyses.

**Results:**

Three axes explained 98.6% of the residualized input variance: PC1 represented a composite body-size/adiposity axis, PC2 a phase angle-anchored body-composition contrast axis, and PC3 an FFMI-dominant axis. The phenotype structure was robust, repeatable, and structurally concordant in NHANES. Within BMI strata, selected associations with estimated VO₂max remained after 24-test BH-FDR correction, including PC2 in Q3 (*β* = 1.82, 95% CI 0.41 to 3.22) and PC3 in Q4 (*β* = 1.72, 95% CI 0.61 to 2.83). For ALT, higher-BMI strata showed divergent PC2 and PC3 associations that remained after BH-FDR correction; for example, in Q4, PC2 was associated with higher ALT (+6.74%, 95% CI 3.23 to 10.38%), whereas PC3 was associated with lower ALT (−6.41%, 95% CI − 10.15% to −2.52%). Expanded and endpoint-specific sensitivity analyses were generally consistent with the primary interpretation, although selected associations were attenuated.

**Conclusion:**

Projected body-composition phenotype axes derived from six routine measurements provided additional explanatory information for estimated VO₂max and ALT within BMI strata. This residualized multivariable framework may help characterize within-BMI heterogeneity in clinical nutrition research, but should not yet be interpreted as a clinical classification or decision-making tool.

## 1 Introduction

Body mass index (BMI) remains the primary entry point for nutritional screening and metabolic risk stratification due to its low cost, reproducibility, and operational efficiency in routine care (1, 2). However, in the field of clinical nutrition, BMI constitutes a size-based index rather than a tissue-resolved phenotype. It does not differentiate between adipose and lean compartments, does not characterize fat distribution, and does not capture qualitative attributes such as cellular integrity or functional reserve (1–3). Consequently, individuals classified within the same BMI category may exhibit substantial variability in central adiposity, muscle preservation, metabolic burden, and physical performance.

The limitations of a single-index approach become more pronounced when outcomes of interest encompass both functional capacity and liver-related metabolic stress. Cardiorespiratory fitness (CRF), commonly indexed by VO₂max, is a clinically meaningful marker, as it reflects integrative physiological reserve and provides prognostic information beyond conventional risk factors (4). Alanine aminotransferase (ALT), although not disease-specific, remains a pragmatic and widely available biomarker of hepatocellular injury in metabolically vulnerable populations (5).

Recent studies using imaging -, impedance -, and multicomponent approaches have demonstrated associations between body-composition characteristics and CRF, as well as frailty (6, 7). Associations have been reported with liver fat, risk of steatotic liver disease, and longitudinal changes in liver disease severity beyond BMI alone (8–10). However, most studies have focused on isolated compartments, single outcomes, or a single measurement platform, rather than on a phenotype framework that can be evaluated across measurement settings (11, 12). Recent reviews and expert guidance have further emphasized that body-composition research requires stronger conceptual integration and clearer methodological standards to support clinically interpretable phenotyping, rather than descriptive compartmentalization alone (11, 12).

In clinical nutrition, this unresolved gap extends beyond methodological considerations and reflects a broader transition from BMI-only classification toward multidimensional risk characterization and clinically anchored definitions of excess adiposity (3, 13). A phenotype framework intended for outpatient use must demonstrate more than cross-sectional associations. It should preserve biologically coherent variation under routine measurement conditions, remain interpretable when input specifications are perturbed, and maintain relevance when applied beyond the derivation setting.

This requirement is particularly important in routine outpatient care, where decisions are based on measurements obtained during standard clinical encounters rather than under tightly controlled research conditions. Therefore, a framework derived from real-world practice and externally projectable to an independent population-based sample would provide a more informative research framework than one restricted to a single analytic context.

In this study, body-composition phenotype axes were derived from six routine measures obtained during real-world outpatient clinical nutrition encounters, using age- and sex-aware preprocessing followed by principal component analysis (PCA). Robustness under alternative input specifications was assessed, repeat-measurement stability was assessed in individuals with serial examinations, and the derived axes were projected into the National Health and Nutrition Examination Survey (NHANES) 1999–2004 for construct-level external evaluation.

To assess clinical relevance across complementary domains, one functional endpoint, estimated VO₂max, and one liver-related biomarker of metabolic stress, ALT, were examined. The extent to which these phenotype axes remained informative within BMI strata was assessed, based on the premise that an informative framework should describe outcome-relevant heterogeneity not resolved by BMI alone.

## 2 Materials and methods

### 2.1 Analytical framework and study cohorts

This study was designed as a derivation-external projection analysis with robustness, sensitivity, repeatability, and diagnostic components. Body-composition phenotype axes were initially derived in a real-world outpatient body-composition cohort and were subsequently projected into NHANES 1999–2004, an independent population-based sample. Within this framework, the outpatient cohort was used to define the latent phenotype space, whereas the NHANES stage was used to evaluate construct-level external projection, structural concordance, and phenotype-outcome associations under a survey-weighted analytic structure (14).

In the outpatient source dataset, 29,900 examination records were screened. The measurement context, data availability, and clinical annotation status of the outpatient derivation cohort are summarized in Supplementary Table S1. Following the application of completeness and plausibility filters, 29,787 valid examinations remained; the only non-zero exclusion was attributable to implausible percent body fat (PBF) values (*n* = 113). These valid examinations corresponded to 23,698 unique individuals. For phenotype derivation, only the earliest valid examination per individual was retained, yielding a derivation cohort of 23,698 individuals. Individuals with at least two valid examinations (*n* = 3,113) comprised the repeat-measurement subset used to assess temporal stability.

For NHANES 1999–2004, 31,126 participant-cycle records were assembled across the relevant source files. As the primary functional endpoint, estimated VO₂max, was derived from the NHANES treadmill fitness component and was available only within the age range covered by that examination framework, the external projection stage was restricted *a priori* to individuals aged 20–49 years (15–18). Following the exclusion of individuals outside this age range or with missing age, and subsequent restriction to those with complete and finite body-composition variables required for projection, the final analytic base sample comprised 5,060 individuals. Outcome-specific complete-case subsets included 2,721 individuals for estimated VO₂max and 4,857 for ALT.

**Figure 1:**
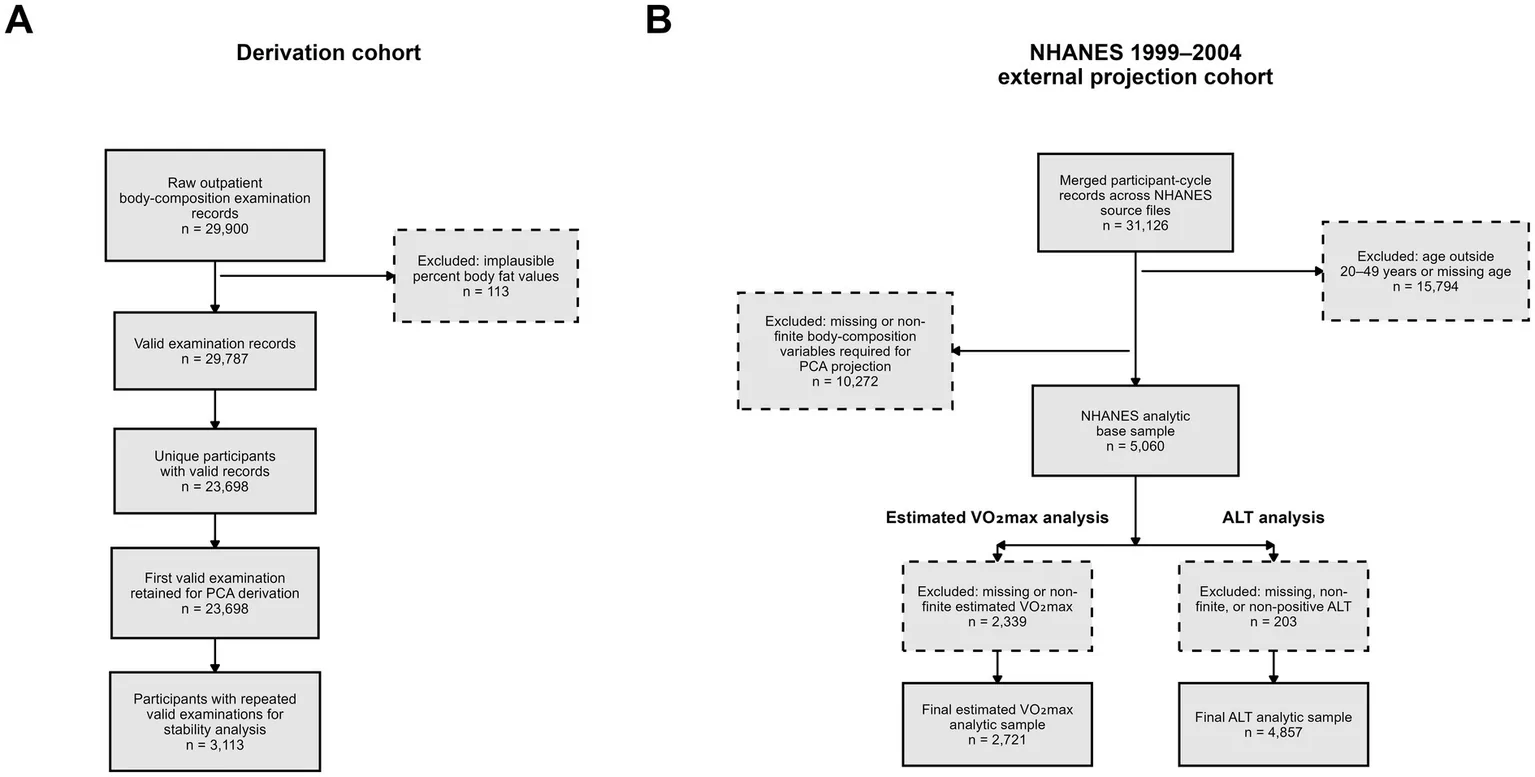
Study flow diagram for the outpatient derivation cohort and NHANES 1999–2004 external projection cohort. **(A)** Illustrates the assembly of the outpatient derivation cohort, including exclusion of records with implausible percent body fat values, retention of the first valid examination per participant for PCA derivation, and selection of the repeat-measurement subset for stability analyses. **(B)** Illustrates the construction of the NHANES analytic base sample from merged participant-cycle records and the derivation of outcome-specific complete-case subsets for estimated VO_2_max and ALT analyses. The NHANES cohort was used for construct-level external projection and phenotype-outcome association analyses rather than for device-level validation of an equivalent clinical tool.

The primary objective was to determine whether projected, age- and sex-residualized body-composition phenotype axes provided explanatory information for estimated VO_2_max and ALT within common BMI strata. Accordingly, the principal NHANES analyses were conducted within survey-weighted unified BMI quartiles defined from the analytic base sample. Robustness analyses, repeatability analyses, and alternative BMI-stratification schemes were retained as supportive analyses. The derivation cohort, the NHANES analytic base sample, and the outcome-specific subsets are summarized in Figure 1.

Ethical approval for the outpatient derivation cohort was granted by the Ethics Committee on Biomedical Research, West China Hospital of Sichuan University (approval No. 2021 Review [No. 752]). All procedures involving human participants were conducted in accordance with the ethical standards of the institutional research committee and with the principles of the Declaration of Helsinki. As retrospectively collected outpatient examination data were used, the requirement for written informed consent was waived by the ethics committee. The outpatient dataset was de-identified prior to analysis, and all analyses were conducted using de-identified data. NHANES 1999–2004 protocols were approved by the National Center for Health Statistics Research Ethics Review Board, and documented informed consent was obtained from all participants. The present NHANES analysis was conducted using publicly available de-identified data.

### 2.2 Body-composition inputs and cross-cohort variable harmonization

In the derivation cohort, body composition was assessed during routine outpatient care using the InBody 770 analyzer (InBody Co., Seoul, Korea), a standing direct segmental multi-frequency bioelectrical impedance platform equipped with octopolar tactile electrodes and proprietary body-composition algorithms (19, 20). Routine device outputs were used to obtain body fat mass, fat-free mass, PBF, and whole-body phase angle (PhA). BMI was calculated from measured weight and height. Waist-to-height ratio (WHtR) was calculated as waist circumference divided by height. Fat mass index (FMI) and fat-free mass index (FFMI) were calculated as body fat mass and fat-free mass, respectively, divided by height squared.

This input set was selected to represent complementary body-composition domains: overall size (BMI), central adiposity (WHtR), adiposity burden (FMI and PBF), lean-tissue reserve (FFMI), and impedance-derived tissue quality (PhA) rather than to restate body size using a single anthropometric surrogate (12, 21, 22).

For external projection in NHANES 1999–2004, instrument-identical measurements were not available. Construct-level harmonization was therefore implemented by mapping each outpatient input to an analogous NHANES variable, assembled from standardized anthropometry and bioelectrical impedance analysis (BIA) components (23–28). Specifically, standing height, body weight, and waist circumference were obtained from the NHANES Body Measures component, whereas bioelectrical variables and derived body-composition outputs were obtained from the NHANES BIA component (23–28).

Analogous projection variables were then constructed as follows: BMI was calculated as weight divided by height squared; WHtR as waist circumference divided by height; FMI and FFMI from NHANES fat mass and fat-free mass normalized to height squared; and PBF from the NHANES PBF variable (26–28). PhA was obtained from the 50-kHz whole-body phase-angle variable when directly available. When this field was unavailable, PhA was derived from 50-kHz reactance and resistance as arctangent (Xc/R) × 180/*π*, consistent with standard impedance derivation (21, 22, 26–28).

Critically, this harmonization strategy was intended to preserve construct correspondence for projection rather than to establish strict measurement equivalence between the outpatient InBody platform and the NHANES examination infrastructure (12, 19, 20). The six variables entered into PCA were BMI, WHtR, FMI, FFMI, PBF, and PhA.

### 2.3 Derivation and external projection of phenotype axes

To extract body-composition covariance in the outpatient derivation cohort, after accounting for demographic structure, each of the six input variables was residualized for age and sex before principal component analysis (PCA) (29, 30). For each input variable, the residualization model included a natural spline of age with three degrees of freedom and an age-by-sex interaction:


Y~ns(age,df=3)×sex

The two internal spline knots were fixed at 31 and 46 years, corresponding to the 33rd and 67th percentiles of age in the derivation cohort. Residualized inputs were then standardized using derivation-cohort reference centering and scaling parameters, and PCA was performed on the standardized residuals. Component signs were oriented so that higher PC1 corresponded to higher WHtR, higher PC2 to higher PhA, and higher PC3 to higher FFMI. The derivation loadings, explained variance, residualization specification, and reference standardization values are reported in Supplementary Table S2.

The external projection step preserved the derivation-defined phenotype basis. In NHANES, the six harmonized body-composition inputs were residualized using the same model form and the derivation-defined internal knot locations of 31 and 46 years. To preserve the derivation-defined phenotype basis while accommodating cohort-specific measurement distributions, the age- and sex-residualization models were refitted within NHANES using the same model form and derivation-defined knot locations. Thus, residualization coefficients were estimated in the external sample, whereas the standardization parameters and PCA loading matrix were carried forward from the derivation cohort. The resulting residualized variables were standardized using the derivation reference parameters and projected onto the fixed derivation rotation matrix. For each participant, projected component scores were calculated as the sum of derivation-standardized residualized inputs weighted by the corresponding derivation loading vector. Accordingly, the projected PC scores represent age- and sex-residualized body-composition phenotype-axis scores rather than raw body-composition values.

Robustness of the phenotype structure was assessed in the derivation cohort by repeating PCA after excluding BMI or WHtR and correlating the resulting scores with those from the six-variable reference solution. Repeatability was assessed in participants with serial outpatient examinations by projecting the first and last valid examinations into the fixed phenotype space and correlating paired scores. Structural concordance in NHANES was assessed by comparing the derivation PCA with an internal NHANES PCA using loading correlations and absolute Tucker congruence coefficients (31), allowing for the sign indeterminacy of PCA. These analyses are reported in Supplementary Tables S3, S4.

Additional residualization diagnostics evaluated the influence of age-knot specification on external projection by comparing the reference projected scores with scores obtained using NHANES-specific internal knots and linear age-by-sex residualization. Score correlations and outcome-association directional concordance diagnostics are reported in Supplementary Table S5.

### 2.4 Ascertainment of estimated VO_2_max

The primary functional endpoint in the NHANES was estimated maximal oxygen uptake (estimated VO₂max). This variable was derived from the NHANES cardiovascular fitness component, which employed a submaximal treadmill protocol rather than direct cardiopulmonary exercise testing (15–18). Treadmill protocols were assigned according to age, sex, BMI, and self-reported physical activity (18), and estimated VO₂max was inferred from heart-rate responses to known submaximal workloads (15–18). The outcome is referred to throughout as estimated VO₂max rather than measured VO₂max.

Estimated VO₂max-specific analyses included participants with non-missing estimated VO₂max, positive examination weights, complete projected phenotype scores, and complete covariates. As the fitness examination was administered only within the treadmill-eligible subsample, individuals with and without available estimated VO₂max were compared descriptively within the NHANES analytic base sample; weighted characteristics are presented in Supplementary Table S6. In addition, protocol-adjusted sensitivity models were fitted using the assigned exercise protocol variable from the cardiovascular fitness component (CVAPROT). Protocol-variable availability and protocol-adjusted sensitivity models are reported in Supplementary Table S7.

These comparisons were used to characterize the selection profile of the fitness subset and were treated as supportive rather than as a formal correction for selection bias.

### 2.5 Ascertainment of ALT

The primary liver-related metabolic endpoint was ALT, obtained from the NHANES standard biochemistry profile (32–34). ALT-specific analyses included individuals with non-missing ALT values, positive examination weights, complete projected phenotype scores, and complete covariates.

As ALT exhibited a right-skewed distribution, regression models were fitted on the natural-log scale. Model coefficients were subsequently exponentiated and expressed as the percent difference in ALT per 1-standard deviation (SD) increment in the corresponding phenotype axis, together with 95% confidence intervals (CI).

To assess the robustness of ALT associations to major liver-related confounding factors, clean-sample sensitivity analyses were conducted after excluding participants with heavy alcohol use or positive hepatitis serology. Primary and expanded clean-sample models are reported in Supplementary Table S8.

### 2.6 Covariates and sensitivity-analysis variables

Overall, demographic covariates included age, sex, and race/ethnicity. Age and sex were used in the residualization step for phenotype derivation and external projection, whereas age, sex, and race/ethnicity were included as covariates in the primary NHANES outcome models.

Additional behavioral and clinical variables were harmonized across NHANES 1999–2004 for expanded covariate-adjusted and sensitivity analyses, following NHANES analytic guidance and cycle-specific questionnaire, laboratory, and prescription-medication documentation (14, 35–43). For estimated VO₂max models, the expanded covariate set included physical activity, smoking status, and recent health status. For ALT models, the expanded covariate set included physical activity, alcohol intake, smoking status, and diabetes status. For ALT clean-sample sensitivity analyses, heavy alcohol use and positive hepatitis serology were used to define the exclusion criteria. Hepatitis-related variables included hepatitis B surface antigen positivity and hepatitis C-related serologic or confirmatory markers, according to availability across the included NHANES cycles. Prescription-medication variables were harmonized for descriptive assessment. Complete-case profiles and harmonized covariate domains are summarized in Supplementary Tables S9, S10.

### 2.7 Statistical analysis

Continuous variables are summarized as mean ± standard deviation or weighted mean ± standard deviation, as appropriate. Categorical variables are summarized as number and percentage or weighted percentage, as appropriate. NHANES analyses incorporated the multistage complex survey design, including examination weights, strata, and primary sampling units (14, 44). For pooled 1999–2004 analyses, six-year examination weights were constructed from the published cycle-specific examination weights according to NHANES analytic guidance (14).

A single set of weighted unified BMI quartiles was defined from the NHANES analytic base sample using Mobile Examination Center examination weights and was applied consistently across estimated VO₂max and ALT analyses. This yielded the following quartile cut-points: 23.16, 26.37, and 30.45 kg/m^2^. Sensitivity analyses using World Health Organization BMI categories and outcome-specific BMI quartiles are reported in Supplementary Tables S11, S12.

Primary outcome associations were estimated using survey-weighted axis-specific regression models fitted within each unified BMI quartile. For estimated VO₂max, models were fitted on the original scale and results are presented as adjusted *β* coefficients with 95% confidence intervals per 1-standard deviation increment in the projected phenotype axis. For ALT, models were fitted on the natural-log scale and results are presented as back-transformed percent differences with 95% confidence intervals per 1-standard deviation increment in the projected phenotype axis. Primary models were adjusted for age, sex, and race/ethnicity. Sequential demographic adjustment models are reported in Supplementary Table S13.

Expanded covariate-adjusted models were fitted as sensitivity analyses. Estimated VO₂max expanded models additionally adjusted for physical activity, smoking status, and recent health status. ALT expanded models additionally adjusted for physical activity, alcohol intake, smoking status, and diabetes status. Expanded model results are reported in Supplementary Table S14.

Multiplicity-aware analyses were conducted using the Benjamini-Hochberg false-discovery-rate procedure (45). The primary correction was applied across the 24 primary stratum-specific tests, defined as three phenotype axes across four BMI quartiles for two outcomes. BMI-stratum × phenotype-axis interaction tests were also performed for each outcome and phenotype axis using design-based global tests, with false-discovery-rate correction across the six interaction tests.

Incremental explanatory information was evaluated by comparing survey-weighted base models with extended models that added PC1, PC2, and PC3. For the overall analysis, the base model included unified BMI quartile, age, sex, and race/ethnicity. Within each BMI quartile, the base model included age, sex, and race/ethnicity. Extended models added the three projected phenotype axes, and incremental information was summarized using weighted model-based *R*^2^, Δ*R*^2^, and joint Wald tests. These multiplicity-aware, interaction, and incremental-information analyses are reported in Supplementary Tables S15–S17.

Supportive diagnostics included PCA suitability diagnostics based on residualized derivation inputs, including pairwise correlations, the Kaiser-Meyer-Olkin measure of sampling adequacy, Bartlett’s test of sphericity (46, 47), and variable-level measures of sampling adequacy. Effective sample-size and weight-variation diagnostics, including Kish effective sample sizes (48) and approximate design effects based on weight variation, were calculated to characterize the reduction in effective information due to unequal survey weighting; these diagnostics were not intended as formal prospective power calculations. Exploratory biochemical screening analyses were retained as supportive analyses. These analyses are reported in Supplementary Tables S18–S20. All statistical tests were two-sided. Analyses were conducted in R version 4.5.2.

## 3 Results

### 3.1 Cohort characteristics

Cohort characteristics are presented in Table 1. Following screening, the derivation dataset comprised 29,787 valid outpatient body-composition examinations, corresponding to 23,698 unique individuals who contributed the first valid measurement to phenotype derivation. In this derivation cohort, the mean age was 40.2 ± 16.1 years, and 14,234 participants (60.1%) were women. The mean BMI was 25.74 ± 5.88 kg/m^2^. The corresponding mean values for the remaining phenotype inputs were 0.54 ± 0.08 for WHtR, 8.86 ± 4.07 kg/m^2^ for FMI, 16.45 ± 2.16 kg/m^2^ for FFMI, 32.13 ± 9.23% for PBF, and 5.25 ± 0.78° for phase angle.

**Table 1:** Characteristics of the derivation cohort and NHANES analytic base sample.

Characteristic	Derivation cohort	NHANES analytic base sample
Study setting	Outpatient body-composition cohort	NHANES 1999–2004
Valid records screened, *n*	29,787	—
Participants contributing to PCA derivation/projection, n	23,698	5,060
Age, years	40.2 ± 16.1	34.6 ± 8.8
Female sex, *n* (%)	14,234 (60.1%)	2,454 (48.5%)
Race/ethnicity, *n* (%)		
Hispanic	—	1,562 (30.9%)
Non-Hispanic Black	—	1,069 (21.1%)
Non-Hispanic White	—	2,229 (44.1%)
Other	—	200 (4.0%)
Body-composition variables used for PCA		
BMI, kg/m^2^	25.74 ± 5.88	27.70 ± 5.95
WHtR	0.54 ± 0.08	0.55 ± 0.09
FMI, kg/m^2^	8.86 ± 4.07	9.03 ± 4.42
FFMI, kg/m^2^	16.45 ± 2.16	18.67 ± 3.67
PBF, %	32.13 ± 9.23	31.60 ± 10.71
Phase angle, °	5.25 ± 0.78	6.96 ± 0.88
Outcome availability		
Estimated VO_2_max available, *n* (%)	—	2,721 (53.8%)
ALT available, *n* (%)	—	4,857 (96.0%)

Data are presented as mean ± SD or *n* (%). NHANES, National Health and Nutrition Examination Survey; BMI, body mass index; WHtR, waist-to-height ratio; FMI, fat mass index; FFMI, fat-free mass index; PBF, percent body fat; ALT, alanine aminotransferase.

The age-restricted NHANES analytic base sample included 5,060 participants aged 20–49 years. Compared with the derivation cohort, this external sample was younger on average (34.6 ± 8.8 years), included a lower proportion of women (48.5%), and exhibited a modestly larger body-size profile, with a mean BMI of 27.70 ± 5.95 kg/m^2^ and a mean FFMI of 18.67 ± 3.67 kg/m^2^. The racial/ethnic composition of the NHANES analytic base sample was 30.9% Hispanic, 21.1% non-Hispanic Black, 44.1% non-Hispanic White, and 4.0% Other. As directly comparable race/ethnicity data were unavailable in the derivation cohort, these distributions are reported for NHANES only.

Within NHANES, outcome availability differed by design. ALT was available for 4,857 participants (96.0%), whereas estimated VO₂max was available for 2,721 participants (53.8%). This asymmetry reflected the treadmill-based fitness examination framework rather than generalized missingness in the analytic base sample. Supplementary Table S6 provides a weighted descriptive comparison of participants with and without estimated VO₂max to characterize the availability profile of the fitness subset, whereas Table 1 summarizes the overall analytic base sample.

### 3.2 Derivation of phenotype axes, PCA diagnostics, robustness, and repeatability

**Figure 2:**
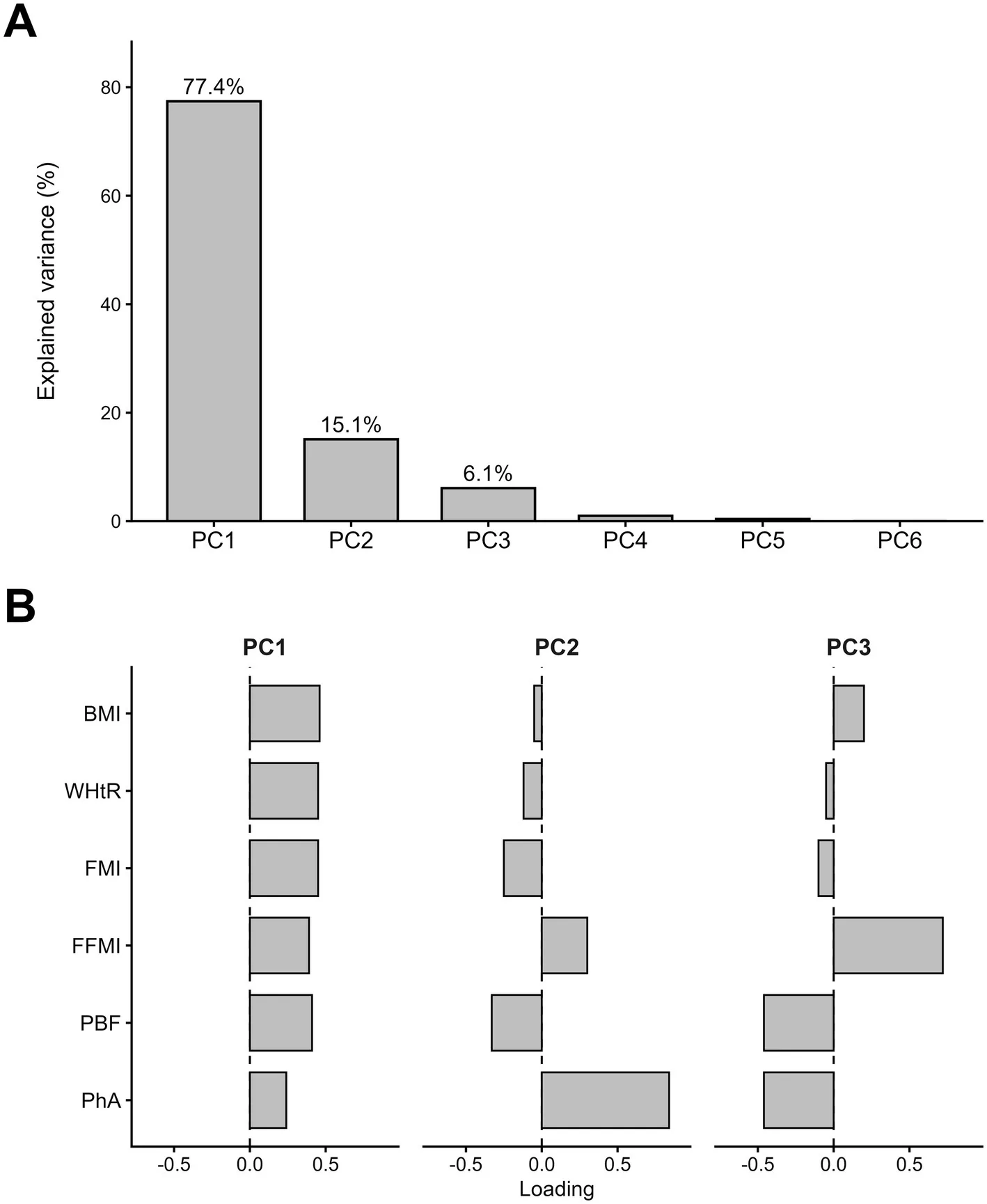
Derivation of body-composition phenotype axes in the outpatient derivation cohort. **(A)** Scree plot depicting the proportion of residualized input variance explained by each principal component. The first three components accounted for 77.4, 15.1, and 6.1% of total variance, respectively, corresponding to a cumulative explained variance of 98.6%. **(B)** Loading plot for the first three components derived from six routine body-composition variables following residualization for age and sex. PC1 demonstrated broadly positive loadings across body-size and body-composition variables and was interpreted as a composite body-size/adiposity axis. PC2 was primarily anchored by phase angle, with a smaller positive contribution from FFMI and inverse contributions from adiposity-related variables, supporting interpretation as a PhA-anchored body-composition contrast axis. PC3 was dominated by FFMI, with inverse contributions from PBF and phase angle, supporting interpretation as an FFMI-dominant axis. The axes should be interpreted as operational summaries of a residualized body-composition covariance structure rather than as direct biological entities or diagnostic categories.

Figure 2 summarizes the variance architecture and loading profile of the six-variable PCA used to derive the phenotype space. The first three principal components explained 77.4%, 15.1%, and 6.1% of total variance, respectively, yielding a cumulative explained variance of 98.6%.

PC1 was characterized by uniformly positive loadings across BMI, WHtR, FMI, FFMI, PBF, and phase angle, consistent with a composite body-size axis. PC2 was most strongly anchored by phase angle, with a smaller positive contribution from FFMI and inverse loadings for adiposity-related variables, supporting its interpretation as a phase angle-anchored body-composition contrast axis. PC3 was dominated by FFMI, with inverse contributions from PBF and phase angle, indicating an FFMI-dominant axis rather than a generic lean-mass surrogate.

PCA suitability diagnostics supported use of a dimensional-reduction approach for the residualized input matrix. The overall Kaiser-Meyer-Olkin measure was 0.776, and Bartlett’s test showed a large departure from an identity correlation matrix (*χ*^2^ = 288,090.08; df = 15; *p* < 0.001). Pairwise correlations among BMI, WHtR, FMI, and PBF were high, consistent with a compact residualized body-composition covariance structure. Detailed correlation, KMO, and Bartlett diagnostics are reported in Supplementary Table S18.

The phenotype structure was highly robust when the input set was perturbed. Following exclusion of BMI, correlations between the reference and sensitivity-derived scores were 0.999, 1.000, and 0.999 for PC1-PC3, respectively. Following exclusion of WHtR, the corresponding correlations were 0.998, 0.999, and 1.000. Repeatability across serial outpatient examinations was maintained. Among individuals with at least two valid visits, correlations between first and last scores were 0.81 for PC1, 0.85 for PC2, and 0.74 for PC3. Exact derivation loadings, explained variance, and robustness/repeatability metrics are provided in Supplementary Tables S2, S4.

These findings indicate that the three-axis phenotype space was statistically compact, suitable for dimensional reduction, robust to modest changes in input composition, and reproducible at the individual level across repeated measurements.

### 3.3 External projection, structural concordance, and residualization diagnostics

When PCA was re-estimated internally in the age-restricted NHANES sample using the same six variables regressed on age and sex, the projected phenotype axes demonstrated good-to-excellent structural concordance with the derivation-defined components after allowance for sign indeterminacy. Loading correlations were 0.978, 0.923, and 0.943 for PC1-PC3, respectively. The corresponding absolute Tucker congruence coefficients were 0.980, 0.925, and 0.923.

These coefficients indicate that the latent body-composition structure defined in the outpatient derivation cohort remained identifiable following projection into the external NHANES sample, although exact loadings were not identical across cohorts. The full structural concordance metrics are presented in Supplementary Table S3.

Residualization diagnostics showed that the projected scores were not materially dependent on the exact age-knot specification used for external projection. Compared with the submitted primary projection using derivation-defined knots at 31 and 46 years, projected scores obtained using NHANES-specific internal knots showed correlations of 1.000, 1.000, and 1.000 for PC1-PC3. Scores obtained using linear age-by-sex residualization showed correlations of 0.999, 0.9954, and 0.9998 for PC1-PC3, respectively. Directional concordance of outcome associations was 12/12 for both estimated VO₂max and ALT under the alternative residualization specifications. These diagnostics are reported in Supplementary Table S5.

### 3.4 Primary associations of projected phenotype axes with estimated VO₂max and ALT within BMI quartiles

**Figure 3:**
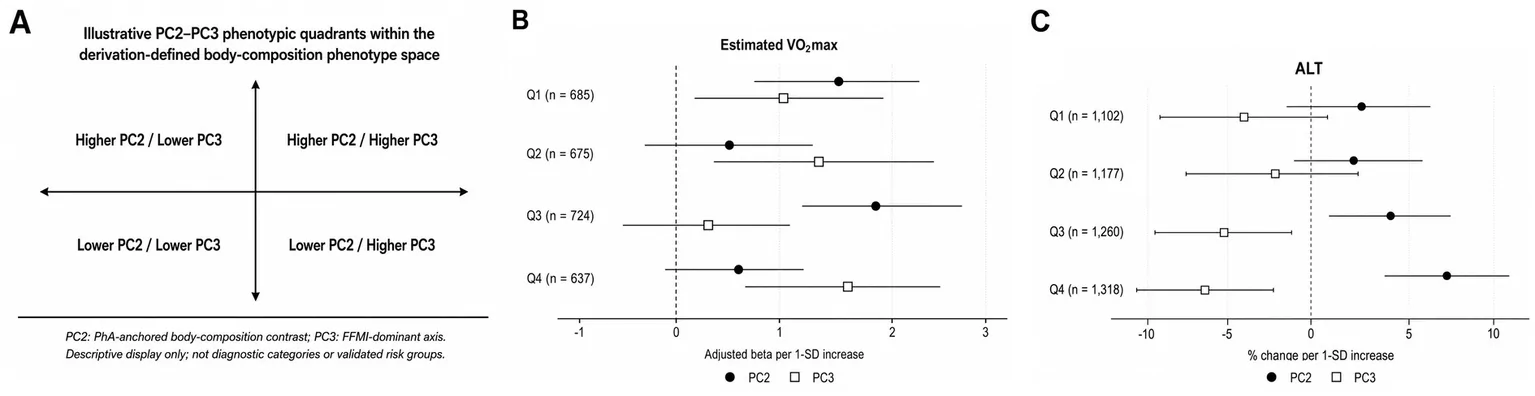
Illustrative PC2-PC3 phenotype space and within-BMI associations of non-size projected phenotype axes with estimated VO_2_max and ALT. **(A)** Shows the illustrative PC2-PC3 phenotype space within the derivation-defined body-composition coordinate system. PC2 is interpreted as a PhA-anchored body-composition contrast axis, and PC3 as an FFMI-dominant axis. The quadrant display is provided for descriptive interpretation only and should not be interpreted as defining diagnostic categories, validated clinical risk groups, or treatment-decision strata. **(B)** Shows survey-weighted associations of projected PC2 and PC3 scores with estimated VO_2_max within unified BMI quartiles in NHANES 1999–2004. Results are presented as adjusted *β* coefficients per 1-SD increment in the corresponding projected phenotype axis, with 95% confidence intervals. **(C)** Shows survey-weighted associations of projected PC2 and PC3 scores with ALT within unified BMI quartiles in NHANES 1999–2004. Results are presented as percent differences in ALT per 1-SD increment in the corresponding projected phenotype axis after model fitting on the natural-log scale and subsequent back-transformation, with 95% confidence intervals. Unified BMI quartiles were defined from the NHANES analytic base sample using MEC examination weights and were applied consistently across outcome analyses. Regression models were adjusted for age, sex, and race/ethnicity. Estimated VO_2_max analyses included individuals with non-missing estimated VO_2_max, positive examination weights, complete projected phenotype scores, and complete covariates; ALT analyses used the corresponding ALT-specific complete-case subset. Focuses on PC2 and PC3 as the non-size phenotype axes displayed in the PC2-PC3 interpretive space; PC1 associations are reported in the corresponding supplementary model tables.

Figure 3 summarizes the within-BMI associations of projected phenotype axes with estimated VO₂max and ALT in NHANES. The PC2-PC3 phenotypic map is provided as an interpretive display of within-BMI body-composition heterogeneity, whereas the forest plots present survey-weighted outcome associations.

For estimated VO₂max, PC1 demonstrated inverse associations, reaching statistical significance in Q1 (*β* = −3.48 per SD, 95% CI − 6.28 to −0.67; *n* = 685) and Q4 (*β* = −2.64, 95% CI − 4.56 to −0.73; *n* = 637). Both associations remained significant after the 24-test BH-FDR correction. In contrast, higher non-size phenotype scores were associated with higher estimated VO₂max in a stratum-specific pattern. PC2 was positively associated with estimated VO₂max in Q1 (*β* = 1.46, 95% CI 0.61–2.30; *n* = 685) and Q3 (β = 1.82, 95% CI 0.41–3.22; *n* = 724), and both associations remained significant after BH-FDR correction. PC3 was positively associated with estimated VO₂max in Q2 and Q4 in the primary models; after BH-FDR correction, the Q4 association remained significant (*β* = 1.72, 95% CI 0.61 to 2.83; *n* = 637), whereas the Q2 association did not. Primary estimated VO₂max associations and FDR results are reported in Supplementary Tables S15, S21.

A similarly differentiated pattern was observed for ALT, modeled on the natural-log scale and expressed as percent difference. PC1 was positively associated with ALT across all quartiles, with effect estimates ranging from +15.6% to +19.1% per SD. These associations remained significant after BH-FDR correction. PC2 and PC3 demonstrated divergent associations in the higher-BMI strata. PC2 was associated with higher ALT in Q3 (+4.06% per SD, 95% CI 0.91 to 7.31%; *n* = 1,260) and Q4 (+6.74%, 95% CI 3.23 to 10.38%; *n* = 1,318), whereas PC3 was associated with lower ALT in Q3 (−5.18%, 95% CI − 8.49% to −1.75%) and Q4 (−6.41%, 95% CI − 10.15% to −2.52%). These Q3 and Q4 PC2/PC3 associations remained significant after BH-FDR correction. Primary ALT associations and FDR results are reported in Supplementary Tables S15, S21.

Sensitivity analyses based on WHO BMI categories and outcome-specific BMI quartiles yielded directionally concordant findings. Under WHO BMI categories, the clearest ALT differentiation was observed in the ≥30 kg/m^2^ group, where PC2 was associated with higher ALT (+6.59% per SD) and PC3 with lower ALT (−6.10% per SD). Primary BMI quartile results are reported in Supplementary Table S21, sequential models in Supplementary Table S13, and sensitivity analyses in Supplementary Tables S11, S12.

Together, these primary analyses indicate that projected phenotype axes captured within-BMI heterogeneity in estimated VO₂max and ALT, with association patterns differing descriptively by axis, outcome, and BMI stratum.

### 3.5 Expanded covariate, interaction, incremental-information, and endpoint-specific sensitivity analyses

Expanded covariate-adjusted models generally preserved the primary association patterns. For estimated VO₂max, the most consistent expanded-model associations were observed for PC2 in Q1 and Q3 and for PC1, PC2, and PC3 in Q4. In Q4, PC1 remained inversely associated with estimated VO₂max (*β* = −2.71, 95% CI − 4.66 to −0.75; *p* = 0.009), whereas PC2 (*β* = 0.91, 95% CI 0.10 to 1.72; *p* = 0.029) and PC3 (*β* = 1.60, 95% CI 0.44 to 2.75; *p* = 0.008) were positively associated with estimated VO₂max. For ALT, PC1 remained positively associated with ALT in Q1 and Q4, and the Q3-Q4 divergence between PC2 and PC3 persisted. In Q4, PC2 was associated with higher ALT (+6.49%, 95% CI 2.60 to 10.53%; *p* = 0.002), whereas PC3 was associated with lower ALT (−6.10%, 95% CI − 10.98% to −0.94%; *p* = 0.023). Expanded covariate-adjusted results are reported in Supplementary Table S14, and complete-case comparisons are reported in Supplementary Table S9.

Formal BMI-stratum × phenotype-axis interaction tests did not provide FDR-supported evidence of statistical interaction. Across the six outcome-axis interaction tests, BH-FDR *q* values were >0.05. Accordingly, the within-BMI association patterns reported above should be interpreted as descriptive stratum-specific patterns rather than as statistically confirmed BMI-stratum effect modification. Interaction results are reported in Supplementary Table S16.

Incremental model comparisons showed that the projected phenotype axes provided additional explanatory information beyond BMI and demographic covariates. In overall models, adding PC1-PC3 increased weighted model-based R^2^ from 0.203 to 0.233 for estimated VO₂max (Δ*R*^2^ = 0.030; Wald *p* < 0.001; BH-FDR *q* < 0.001) and from 0.248 to 0.262 for ALT (Δ*R*^2^ = 0.014; Wald *p* < 0.001; BH-FDR *q* < 0.001). Within BMI quartiles, Δ*R*^2^ values ranged from 0.022 to 0.051 for estimated VO₂max and from 0.009 to 0.032 for ALT. Incremental model-comparison results are reported in Supplementary Table S17.

Endpoint-specific sensitivity analyses were generally consistent with the main interpretation. For estimated VO₂max, protocol-adjusted sensitivity models were fitted using the assigned treadmill protocol variable. After combined expanded covariate and protocol adjustment, the most consistent associations were observed for PC2 in Q3 and for PC1, PC2, and PC3 in Q4. In Q4, PC1 remained inversely associated with estimated VO₂max (*β* = −3.57, 95% CI − 5.75 to −1.39; *p* = 0.002), whereas PC2 (*β* = 1.03, 95% CI 0.18 to 1.87; *p* = 0.019) and PC3 (*β* = 1.46, 95% CI 0.25 to 2.67; *p* = 0.020) remained positively associated. Protocol-variable availability and protocol-adjusted models are reported in Supplementary Table S7.

For ALT, clean-sample sensitivity analyses excluded participants with heavy alcohol use or positive hepatitis serology. In the primary clean-sample model, PC1 remained positively associated with ALT in Q1, Q3, and Q4; PC2 remained positively associated with ALT in Q4; and PC3 remained inversely associated with ALT in Q3 and Q4. In the expanded clean-sample model, PC1 remained positively associated with ALT in Q1, Q3, and Q4, and PC2 remained positively associated with ALT in Q3 and Q4. The PC3 associations were directionally similar but attenuated in the expanded clean-sample model. These analyses are reported in Supplementary Table S8.

Effective sample-size diagnostics indicated that unequal survey weighting reduced the effective information relative to nominal sample sizes but preserved analyzable outcome-specific samples. The estimated VO₂max analytic subset had a nominal n of 2,721 and a Kish effective n of 1,996, whereas the ALT analytic subset had a nominal n of 4,857 and a Kish effective n of 3,452. BMI-quartile-specific diagnostics are reported in Supplementary Table S19. Covariate harmonization for expanded and clean-sample sensitivity analyses is summarized in Supplementary Table S10.

## 4 Discussion

### 4.1 Principal findings

A parsimonious body-composition phenotype framework was derived from six routine measurements in an outpatient cohort, and the extent to which the resulting axes remained informative following conventional BMI grouping was subsequently examined. The principal finding was that projected, age- and sex-residualized phenotype axes provided additional explanatory information for estimated VO₂max and ALT within BMI strata. This pattern was particularly evident for ALT in higher-BMI strata, where non-size axes showed divergent associations, and was also observed for estimated VO₂max in selected strata. This contrast highlights heterogeneity that may be overlooked in routine practice, where BMI often serves as the primary interpretive framework. These findings do not indicate that BMI lacks clinical utility; rather, they suggest that body-composition information routinely obtained in nutritional and outpatient assessment can be organized into a residualized multivariable phenotype framework that helps describe functional and liver-enzyme heterogeneity not captured by BMI alone.

### 4.2 Interpretation of phenotype axes

The three axes were interpretable; however, none should be considered a direct surrogate for a single measured trait. PC1 functioned as a composite body-size dimension, reflecting shared variance across indices related to both overall size and adiposity. PC2 loaded most strongly on phase angle and was concurrently aligned with a smaller positive contribution from FFMI and inverse contributions from adiposity-related variables. It is therefore most appropriately described as a PhA-anchored body-composition contrast axis. PC3, in contrast, was dominated by FFMI and is therefore best interpreted as an FFMI-dominant axis. However, describing it as a simple lean-mass axis would overstate its specificity, as meaningful covariance with the broader body-composition space was retained. These labels should therefore be regarded as operational summaries of data-derived constructs rather than direct biological entities (12, 21, 22).

This distinction is methodologically and biologically relevant. PCA suitability diagnostics supported the use of dimensional reduction, but the high correlations among BMI, WHtR, FMI, and PBF indicate that the derived axes summarize a compact residualized body-composition covariance structure rather than independent biological dimensions. The axis labels should therefore be regarded as operational summaries of data-derived constructs, not as direct biological entities. Recent guidance in body-composition research has cautioned against collapsing adiposity, lean reserve, and impedance-derived tissue properties into interchangeable shorthand when these domains coexist within the same analytic space (12, 19, 20).

Phase angle is often interpreted in relation to cellular integrity, hydration distribution, and muscle-related quality (21, 22). In the present study, however, phase angle entered a multivariable residualized coordinate system rather than being analyzed as an isolated marker. Its contribution should therefore be understood as part of an integrated body-composition contrast. This distinction helps explain why a PhA-anchored axis may show complex and outcome-specific associations with estimated VO₂max and ALT across BMI strata.

### 4.3 Added explanatory information beyond BMI

The practical implication is not that BMI should be discarded. In clinical nutrition, BMI remains useful for screening, broad categorization, and initial risk approximation. However, its utility becomes more limited when applied to more detailed characterization, particularly when the objective is to define phenotype at the individual level or to explain why patients with similar BMI may present differently during routine nutritional assessment (1–3, 13). Within the outpatient setting, such discordance is commonly observed: patients with similar BMI may differ substantially in lean reserve, adiposity distribution, and functional capacity. The present findings provide a structured framework for this observation rather than merely restating it.

This interpretation is consistent with a growing body of literature indicating that obesity evaluation and body-composition assessment should extend beyond BMI as a stand-alone descriptor (1–3, 13). The limitation is not only that BMI may inadequately capture adiposity, but that it may obscure meaningful heterogeneity within its own categories, heterogeneity that may be relevant for physical function and liver-related metabolic burden. Recent evidence indicating that PBF may outperform BMI in mortality prediction among individuals aged 20–49 years, together with outcome-based PBF thresholds that may more directly capture clinically relevant adiposity, supports this perspective (49, 50). Recent evidence on resting energy expenditure prediction also supports the need to interpret fat-free mass, fat mass, age, sex, and physical activity jointly when body-composition phenotypes are used to characterize metabolic profiles (51).

However, caution remains necessary. Adding PC1-PC3 to BMI and demographic covariates increased model-based explanatory information for both estimated VO₂max and ALT. However, the magnitude of these increments was modest. Accordingly, the results support additional explanatory information beyond BMI, but they do not establish superior clinical prediction, risk reclassification, or treatment-selection utility. No phenotype thresholds were derived, and no decision rules were proposed.

The use of weighted unified BMI quartiles also served a specific analytic purpose. These quartiles provided a common BMI reference structure across estimated VO₂max and ALT analyses, reducing the influence of endpoint-specific sample composition and missingness on between-outcome comparisons. Sensitivity analyses using WHO BMI categories and outcome-specific quartiles yielded directionally concordant findings, supporting the stability of the main descriptive patterns. The quartiles should therefore be interpreted as a pragmatic analytic framework rather than as biologically privileged categories.

Related studies have reached similar conclusions using different methodological approaches. Multivariable computed tomography-derived body-composition scores have demonstrated associations with frailty and postoperative outcomes that are not fully explained by BMI, with muscle quantity and quality often providing more consistent signal than global size metrics (7). A recent systematic review and meta-analysis of load-capacity indices concluded that body-composition configurations integrating metabolic load and metabolic capacity are associated with adverse cardiometabolic outcomes (11). In this context, this study extends the literature in a pragmatic direction: phenotype axes were derived from measurements routinely available in outpatient practice, remained structurally identifiable in an external survey sample, and continued to stratify outcome variation within the same BMI stratum (7, 11, 13).

### 4.4 Functional and liver-enzyme heterogeneity

The estimated VO₂max findings provide supportive evidence that the projected phenotype axes are related to a functional endpoint. PC1 was generally inversely associated with estimated VO₂max, consistent with the interpretation that a composite body-size/adiposity dimension is linked to lower estimated functional capacity. Selected non-size axes, particularly PC2 and PC3 in several strata, showed positive associations with estimated VO₂max. Protocol-adjusted sensitivity analyses showed generally consistent patterns after adjustment for the assigned treadmill protocol variable, with the most consistent sensitivity signal observed in the highest BMI quartile.

These findings are compatible with the concept that body-composition structure carries functional information beyond size alone. However, estimated VO₂max in NHANES is a protocol-derived submaximal treadmill estimate rather than directly measured cardiorespiratory fitness. Protocol assignment also incorporated participant characteristics such as BMI and physical activity. Therefore, the estimated VO₂max results should be interpreted as supportive functional evidence rather than definitive validation against directly measured physiological capacity.

Alanine aminotransferase provided a complementary biochemical perspective. PC1 was positively associated with ALT across BMI strata, consistent with a body-size and adiposity-related signal. More informative, however, was the divergence between PC2 and PC3 in higher-BMI strata. In these strata, PC2 tended to be associated with higher ALT, whereas PC3 tended to be associated with lower ALT. This contrast suggests that liver-enzyme heterogeneity may persist among individuals who are similar by BMI but differ in residualized body-composition structure.

Clean-sample analyses excluding heavy alcohol use and positive hepatitis serology supported the general direction of the ALT findings. However, expanded clean-sample models showed attenuation of selected PC3 associations. Therefore, the most stable ALT finding is the higher-BMI association between PC2 and higher ALT, together with the overall directional contrast between PC2 and PC3, rather than uniform robustness of every axis-specific estimate across all sensitivity models.

These findings align with broader evidence that liver-related metabolic risk is shaped by body-composition patterning rather than by BMI alone. Prior studies have linked liver fat, adiposity, muscle quality, and cardiorespiratory capacity in metabolically at-risk populations, and intervention studies suggest that improvements in body composition may accompany changes in liver-related outcomes (8–10, 52–55). The present study extends this literature in a pragmatic direction by showing that a routine-measurement-based phenotype framework can identify explanatory heterogeneity in ALT after BMI grouping has already been applied. The findings should not be interpreted as positioning ALT as a definitive liver endpoint, but they suggest that residualized body-composition structure may help describe liver-enzyme variation within BMI categories that may otherwise appear clinically homogeneous.

### 4.5 Strengths and implications for future research

Several features strengthen this study. First, the phenotype framework was derived from routine body-composition measurements that are already available in many clinical nutrition and outpatient assessment settings, supporting translational relevance. Second, the derivation-to-projection sequence was implemented transparently: derivation-defined age-knot locations, standardization parameters, and the PCA loading matrix were carried into NHANES, while age- and sex-residualization models were refitted in NHANES using the same model form. Third, the phenotype structure was robust to input perturbation, repeatable across serial outpatient measurements, and structurally concordant in the external population-based sample. Fourth, the revised analyses further strengthened interpretability by incorporating multiplicity-aware testing, expanded covariate adjustment, interaction assessment, endpoint-specific sensitivity analyses, PCA suitability diagnostics, and survey-design diagnostics.

Future work should move from explanatory association toward validation and implementation. Prospective studies are needed to determine whether these phenotype axes predict longitudinal clinical outcomes beyond standard anthropometric and body-composition measures. Studies incorporating directly measured cardiorespiratory fitness, imaging-based liver fat, magnetic resonance or dual-energy X-ray absorptiometry body-composition measures, and hard cardiometabolic endpoints would help clarify the physiological interpretation of the axes. Device-calibration studies are also required to evaluate whether similar phenotype coordinates can be transported across different bioelectrical impedance platforms. Finally, intervention studies could assess whether changes in these axes track response to dietary, exercise, pharmacologic, or combined lifestyle interventions.

### 4.6 Limitations

Several limitations should be considered. First, the analyses were cross-sectional. The observed associations between projected phenotype axes, estimated VO₂max, and ALT should therefore not be interpreted as causal relationships. Longitudinal and intervention studies are required to determine whether movement along these axes corresponds to changes in functional or metabolic risk.

Second, the outpatient derivation cohort was a routine body-composition examination cohort rather than a disease-annotated clinical research cohort. Structured information on diagnoses, medication exposure, fasting status, hydration status, lifestyle factors, and specific examination indications was not uniformly available across the full derivation cohort. The derivation stage should therefore be interpreted as construction of a body-composition covariance space, not as derivation of disease-specific clinical categories.

Third, external projection into NHANES was performed at the construct level. The outpatient cohort used the InBody 770 platform, whereas NHANES relied on its own anthropometric and bioelectrical impedance examination infrastructure. Although structural concordance was good to excellent, this does not establish device-level equivalence. Differences in frequency architecture, electrode configuration, raw-data availability, embedded algorithms, and prediction equations may influence body-composition outputs across platforms (19, 20, 56, 57).

Fourth, estimated VO₂max was available only in the NHANES treadmill-eligible subset and was derived from a submaximal treadmill protocol. Protocol assignment incorporated participant characteristics, including BMI and self-reported physical activity, which may introduce dependency between body-size-related phenotype information and the estimated VO₂max outcome. Protocol-adjusted sensitivity analyses reduced, but did not eliminate, this concern and cannot substitute for directly measured cardiorespiratory fitness. Accordingly, estimated VO₂max should be interpreted as a supportive protocol-derived functional endpoint rather than a direct measure of physiological capacity.

Fifth, ALT is not a definitive marker of steatotic liver disease or liver pathology. ALT may be influenced by alcohol exposure, viral hepatitis, medication use, and other hepatic or systemic conditions (5). Clean-sample analyses excluding heavy alcohol use and positive hepatitis serology were performed, but residual liver-related confounding cannot be excluded. Drug-specific hepatotoxic medication exclusion was not implemented because reliable harmonization of medication classes across cycles was not feasible from the available public-use variables.

Sixth, the phenotype axes were residualized and projected constructs rather than raw body-composition values. Their interpretation depends on the residualization, standardization, and projection framework. Although knot-sensitivity analyses showed high score concordance under alternative residualization specifications, the resulting scores should still be interpreted as age- and sex-residualized phenotype-axis values.

Seventh, effective sample-size and weight-variation diagnostics were provided to characterize the reduction in effective information due to unequal NHANES examination weights. However, these diagnostics were not coefficient-specific survey design effects, formal prospective power calculations, or minimum detectable effect estimates. Coefficient-specific minimum detectable effects were not estimated for each survey-weighted stratum-specific model; therefore, weaker stratum-specific associations, particularly those not retained after multiplicity correction or sensitivity analyses, should be interpreted cautiously.

Finally, the present study did not establish clinical prediction, decision thresholds, or treatment-selection utility. The incremental explanatory information beyond BMI was statistically significant but modest. The framework should therefore be considered an explanatory approach for characterizing within-BMI heterogeneity, not a standalone clinical classification tool.

## 5 Conclusion

Projected body-composition phenotype axes derived from six routine measurements provided additional explanatory information for estimated VO₂max and ALT within BMI strata. The projected axes highlighted residualized body-composition heterogeneity not captured by BMI alone, particularly the divergent ALT associations of the PhA-anchored body-composition contrast axis and the FFMI-dominant axis in higher-BMI strata. These findings support the use of multivariable body-composition phenotyping as an explanatory framework for characterizing within-BMI heterogeneity in clinical nutrition research. Prospective, device-calibrated, and intervention-based studies are needed before clinical thresholds or implementation pathways can be established.

## Data Availability

The original contributions presented in the study are included in the article/Supplementary material, further inquiries can be directed to the corresponding author/s.

## Glossary

BMI: body mass index
WHtR: waist-to-height ratio
FMI: fat mass index
FFMI: fat-free mass index
PBF: percent body fat
PhA: phase angle
PCA: principal component analysis
PC1: principal component 1
PC2: principal component 2
PC3: principal component 3
ALT: alanine aminotransferase
NHANES: National Health and Nutrition Examination Survey
Estimated VO_2_max: estimated maximal oxygen uptake
CRF: cardiorespiratory fitness
MASLD: metabolic dysfunction-associated steatotic liver disease
BIA: bioelectrical impedance analysis
WHO: World Health Organization
MEC: Mobile Examination Center
SD: standard deviation
CI: confidence interval
