# Supplementary material for: Body-composition phenotype axes provide additional explanatory information for cardiorespiratory fitness and liver-related metabolic risk within BMI strata
Source: Front Nutr. 2026 Aug 31;13:1881228. doi: 10.3389/fnut.2026.1881228 (PMC13572601; doi:10.3389/fnut.2026.1881228)
Supplement: Supplementary file 1 [file Data_Sheet_1.ZIP › Supplementary Table S21.docx]

**Supplementary Table S21.** Primary survey-weighted associations of projected phenotype axes with estimated VO_2_max and ALT within weighted unified BMI quartiles.

**Panel A.** Weighted unified BMI quartile cutpoints used across primary outcome analyses.

| Cutpoint framework | Q1 range | Q2 range | Q3 range | Q4 range | Q1 cutoff | Q2 cutoff | Q3 cutoff |
| --- | --- | --- | --- | --- | --- | --- | --- |
| Weighted unified quartiles (analytic base sample) | BMI ≤ 23.16kg/m² | 23.16kg/m² < BMI ≤ 26.37kg/m² | 26.37kg/m² < BMI ≤ 30.45kg/m² | BMI > 30.45kg/m² | 23.16kg/m² | 26.37kg/m² | 30.45kg/m² |

**Panel B.** Estimated VO_2_max: adjusted β coefficients per 1-SD increment in projected phenotype axis.

| BMI quartile | n | PC1 effect (95% CI) | PC1 p | PC2 effect (95% CI) | PC2 p | PC3 effect (95% CI) | PC3 p |
| --- | --- | --- | --- | --- | --- | --- | --- |
| Q1 | 685 | -3.48 (-6.28 to -0.67) | 0.020 | 1.46 (0.61 to 2.30) | 0.002 | 1.06 (-0.04 to 2.16) | 0.068 |
| Q2 | 675 | -1.52 (-4.66 to 1.62) | 0.350 | 0.44 (-0.41 to 1.30) | 0.314 | 1.37 (0.16 to 2.59) | 0.033 |
| Q3 | 724 | -2.81 (-5.83 to 0.20) | 0.076 | 1.82 (0.41 to 3.22) | 0.016 | 0.35 (-0.58 to 1.29) | 0.465 |
| Q4 | 637 | -2.64 (-4.56 to -0.73) | 0.010 | 0.58 (-0.15 to 1.30) | 0.127 | 1.72 (0.61 to 2.83) | 0.004 |

**Panel C.** ALT: percent differences per 1-SD increment in projected phenotype axis.

| BMI quartile | n | PC1 effect (95% CI) | PC1 p | PC2 effect (95% CI) | PC2 p | PC3 effect (95% CI) | PC3 p |
| --- | --- | --- | --- | --- | --- | --- | --- |
| Q1 | 1102 | 19.08% (7.93% to 31.37%) | 0.001 | 1.80% (-2.05% to 5.80%) | 0.370 | -3.57% (-9.06% to 2.25%) | 0.232 |
| Q2 | 1177 | 15.62% (2.56% to 30.35%) | 0.023 | 1.67% (-1.94% to 5.41%) | 0.376 | -2.74% (-7.72% to 2.50%) | 0.306 |
| Q3 | 1260 | 17.21% (3.05% to 33.31%) | 0.021 | 4.06% (0.91% to 7.31%) | 0.016 | -5.18% (-8.49% to -1.75%) | 0.006 |
| Q4 | 1318 | 17.40% (11.61% to 23.49%) | <0.001 | 6.74% (3.23% to 10.38%) | <0.001 | -6.41% (-10.15% to -2.52%) | 0.003 |

**Note:** Panel A reports the weighted unified BMI quartiles defined once from the NHANES analytic base sample using MEC examination weights and applied consistently across the primary outcome analyses. Panels B and C report results from survey-weighted regression models adjusted for age, sex, and race/ethnicity. For estimated VO_2_max, effects are shown as adjusted β coefficients with 95% confidence intervals per 1-SD increment in the corresponding projected phenotype axis. For ALT, models were fitted on the natural-log scale and back-transformed; effects are therefore shown as percent differences with 95% confidence intervals per 1-SD increment in the corresponding projected phenotype axis. Projected phenotype axes were derived by external projection of NHANES data into the derivation-defined phenotype space. ALT, alanine aminotransferase.
