# Supplementary material for: Body-composition phenotype axes provide additional explanatory information for cardiorespiratory fitness and liver-related metabolic risk within BMI strata
Source: Front Nutr. 2026 Aug 31;13:1881228. doi: 10.3389/fnut.2026.1881228 (PMC13572601; doi:10.3389/fnut.2026.1881228)
Supplement: Supplementary file 1 [file Data_Sheet_1.ZIP › Supplementary Table S1.docx]

**Supplementary Table S1.** Data availability, measurement context, and clinical annotation status in the outpatient derivation cohort

Panel A. Source cohort, measurement context, and analytic use

| Item | Information |
| --- | --- |
| Source setting | Routine outpatient body-composition examination records from the Department of Clinical Nutrition, West China Hospital, Sichuan University. |
| Measurement context | Routine outpatient body-composition assessment rather than a disease-specific research cohort. |
| Measurement platform | InBody 770, a standing direct segmental multifrequency bioelectrical impedance analysis platform. |
| Raw records screened | 29,900 outpatient body-composition examination records. |
| Valid examination records | 29,787 records after completeness and plausibility screening. |
| Main non-zero exclusion | Implausible percent body fat values, n = 113. |
| Unique participants retained for phenotype derivation | 23,698 individuals, with the first valid examination retained for principal component analysis. |
| Repeat-measurement subset | 3,113 participants with repeated valid examinations for stability analysis. |
| Primary analytic role | Derivation of an age- and sex-residualized body-composition covariance structure from routine body-composition measurements. |
| Ethics and consent | Approved by the Ethics Committee on Biomedical Research, West China Hospital of Sichuan University (approval No. 2021 Review [No. 752]); the requirement for written informed consent was waived for the retrospective de-identified outpatient dataset. |

Panel B. Available and unavailable data domains in the outpatient derivation cohort

| Data domain | Availability in the outpatient derivation database | Used in derivation or internal analyses? | Handling and rationale |
| --- | --- | --- | --- |
| Age and sex | Available | Yes | Used for residualization of each body-composition input before PCA. |
| Anthropometry | Available | Yes | Height, weight, and waist circumference were used to calculate BMI and WHtR. |
| Bioelectrical impedance-derived body-composition outputs | Available | Yes | Routine device outputs supported FMI, FFMI, PBF, and phase angle calculation or extraction. |
| Six phenotype-derivation inputs | Available | Yes | BMI, WHtR, FMI, FFMI, PBF, and PhA formed the predefined input set for PCA. |
| Repeated body-composition examinations | Available for a subset | Yes | Used only for repeat-measurement stability analysis. |
| Disease diagnosis and comorbidity information | Not uniformly structured or reproducibly available across the full source database | No | Not used for disease-specific subgrouping or covariate adjustment in the derivation cohort. |
| Medication exposure | Not uniformly structured or reproducibly available across the full source database | No | Not used for covariate adjustment or medication-specific exclusion in the derivation cohort. |
| Lifestyle factors, including physical activity, alcohol use, and smoking | Not collected as standardized research variables in the outpatient derivation database | No | Not used in the derivation cohort; these domains were addressed in NHANES-based sensitivity analyses where harmonized questionnaire data were available. |
| Fasting status before BIA | Not uniformly available | No | Not used for adjustment or exclusion because standardized fasting information was not consistently captured. |
| Hydration status or recent fluid balance | Not uniformly available | No | Not used for adjustment or exclusion; this was treated as a limitation of routine-care body-composition data. |
| Specific examination indication | Not uniformly structured | No | The derivation cohort was interpreted as a routine outpatient body-composition examination cohort rather than as a disease-indication cohort. |
| Outcome measures such as estimated VO2max or ALT | Not available in the outpatient derivation database | No | Outcome associations were evaluated only in NHANES after external construct-level projection. |
| Missing-data handling | Completeness and plausibility filters were applied to body-composition records | Yes, for analytic eligibility | No imputation was applied for unavailable clinical annotations; analyses were restricted to valid body-composition examinations. |

Panel C. How limited clinical annotation was handled in the revised interpretation

| Issue raised by clinical annotation limits | Handling in the revised analysis | Implication for interpretation |
| --- | --- | --- |
| Incomplete diagnosis/comorbidity annotation | No disease-specific phenotype derivation or disease-subgroup analysis was performed in the outpatient cohort. | The derived axes should be interpreted as body-composition covariance constructs, not as diagnosis-informed clinical categories. |
| Incomplete medication and lifestyle annotation | These domains were not incorporated into outpatient phenotype derivation. Expanded covariate and clean-sample analyses were instead performed in NHANES, where standardized questionnaire and laboratory modules were available. | Residual confounding in the derivation setting cannot be excluded, but the derivation stage did not model clinical outcomes. |
| Unavailable fasting and hydration information | No exclusion or adjustment based on fasting or hydration status was applied in the outpatient cohort. | This supports a pragmatic routine-care interpretation but limits controlled-measurement reproducibility. |
| Routine outpatient rather than disease-specific source population | The outpatient cohort was used only to define the phenotype space; outcome associations and behavioral/clinical sensitivity analyses were evaluated in NHANES. | External projection findings should be interpreted at the construct level and should not be conflated with validation of a disease-specific clinical decision tool. |

**Note:** This table summarizes the data context and clinical annotation status of the outpatient derivation cohort. The cohort was assembled from routine outpatient body-composition examination records and was used to derive a body-composition covariance structure from the predefined six-variable input set. Structured information on diagnoses, comorbidities, medication exposure, lifestyle factors, fasting status, hydration status, and specific examination indications was not uniformly available across the full derivation cohort and was therefore not used for disease-specific subgrouping or covariate adjustment. Accordingly, the derivation cohort should be interpreted as a routine body-composition measurement cohort rather than as a disease-annotated clinical cohort. Outcome associations, expanded behavioral and clinical covariate adjustment, and ALT clean-sample sensitivity analyses were conducted in NHANES.

Abbreviations: ALT, alanine aminotransferase; BIA, bioelectrical impedance analysis; BMI, body mass index; FFMI, fat-free mass index; FMI, fat mass index; NHANES, National Health and Nutrition Examination Survey; PCA, principal component analysis; PBF, percent body fat; PhA, phase angle; Estimated VO₂max, estimated maximal oxygen uptake; WHtR, waist-to-height ratio.
