# Supplementary material for: Body-composition phenotype axes provide additional explanatory information for cardiorespiratory fitness and liver-related metabolic risk within BMI strata
Source: Front Nutr. 2026 Aug 31;13:1881228. doi: 10.3389/fnut.2026.1881228 (PMC13572601; doi:10.3389/fnut.2026.1881228)
Supplement: Supplementary file 1 [file Data_Sheet_1.ZIP › Supplementary Table S2.docx]

**Supplementary Table S2.** Exact derivation PCA parameters and reference standardization values used for external projection.

**Panel A.** PCA loading matrix in the derivation cohort (standardized residualized inputs).

| Variable | PC1 | PC2 | PC3 |
| --- | --- | --- | --- |
| BMI | 0.4589 | -0.0542 | 0.1984 |
| WHtR | 0.4524 | -0.1214 | -0.0585 |
| FMI | 0.4473 | -0.2403 | -0.0991 |
| FFMI | 0.3936 | 0.3056 | 0.7198 |
| PBF | 0.4150 | -0.3301 | -0.4638 |
| PhA | 0.2400 | 0.8498 | -0.4627 |

**Panel B.** Explained variance and eigenvalues (derivation cohort).

| PC | Eigenvalue | Explained variance (%) | Cumulative variance (%) |
| --- | --- | --- | --- |
| PC1 | 4.6463 | 77.44 | 77.44 |
| PC2 | 0.9053 | 15.09 | 92.53 |
| PC3 | 0.3656 | 6.09 | 98.62 |
| PC4 | 0.0492 | 0.82 | 99.44 |
| PC5 | 0.0335 | 0.56 | 100.00 |
| PC6 | 0.0000 | 0.00 | 100.00 |

**Panel C.** Residualization specification (applied to each input prior to PCA and projection).

| Parameter | Specification |
| --- | --- |
| Age term | Natural spline of age (df = 3) |
| Internal knots | 31 and 46 years |
| Knot derivation | 33rd and 67th percentiles of age in the derivation cohort (n = 23,698) |
| Interaction term | Age-by-sex interaction |
| Model formula | Y ~ ns (Age, df = 3) * Sex |
| Component orientation | Signs oriented such that higher PC1 corresponds to higher WHtR, higher PC2 to higher PhA, and higher PC3 to higher FFMI |

**Panel D.** Reference means and standard deviations of residualized inputs used for standardization and external projection.

| Variable | Residual mean (derivation) | Residual SD (derivation) |
| --- | --- | --- |
| BMI | 0.000000 | 5.640298 |
| WHtR | 0.000000 | 0.083455 |
| FMI | 0.000000 | 3.941550 |
| FFMI | 0.000000 | 2.097924 |
| PBF | 0.000000 | 8.450771 |
| PhA | 0.000000 | 0.602557 |

**Note:** PCA was performed on six body-composition variables after residualization for age and sex using a natural spline for age (df = 3) with fixed internal knots at 31 and 46 years and an age-by-sex interaction. Residualized variables were standardized using derivation-cohort reference means and standard deviations before PCA and before external projection. Reference means and standard deviations in Panel D correspond to age- and sex-residualized input variables prior to standardization and are therefore centered at 0 by construction. Component signs were oriented such that higher PC1 corresponds to higher WHtR, higher PC2 to higher PhA, and higher PC3 to higher FFMI.

WHtR, waist-to-height ratio; FMI, fat mass index; FFMI, fat-free mass index; PBF, percent body fat; PhA, phase angle.
