# Supplementary material for: Body-composition phenotype axes provide additional explanatory information for cardiorespiratory fitness and liver-related metabolic risk within BMI strata
Source: Front Nutr. 2026 Aug 31;13:1881228. doi: 10.3389/fnut.2026.1881228 (PMC13572601; doi:10.3389/fnut.2026.1881228)
Supplement: Supplementary file 1 [file Data_Sheet_1.ZIP › Supplementary Table S3.docx]

**Supplementary Table S3.** Structural concordance between the derivation-cohort PCA and the internal NHANES PCA.

| PC | Loadings correlation | Tucker congruence (raw) | Tucker congruence (absolute) | Interpretation |
| --- | --- | --- | --- | --- |
| PC1 | 0.9778 | 0.9796 | 0.9796 | Excellent (>0.95) |
| PC2 | 0.9229 | 0.9249 | 0.9249 | Good (0.85–0.95) |
| PC3 | 0.9430 | 0.9229 | 0.9229 | Good (0.85–0.95) |

**Note:** Structural concordance was assessed by comparing the derivation-cohort PCA with an internal PCA re-estimated in the age-restricted NHANES analytic base sample using the same six residualized body-composition variables. Loadings correlation denotes the Pearson correlation between corresponding loading vectors. Tucker congruence coefficients summarize factor similarity, with values >0.95 interpreted as excellent and 0.85–0.95 as good. Absolute Tucker congruence is reported to allow for the arbitrary sign indeterminacy of PCA. Together, these results support good-to-excellent external recognizability of the derivation-defined phenotype structure in NHANES.
