# Supplementary material for: Body-composition phenotype axes provide additional explanatory information for cardiorespiratory fitness and liver-related metabolic risk within BMI strata
Source: Front Nutr. 2026 Aug 31;13:1881228. doi: 10.3389/fnut.2026.1881228 (PMC13572601; doi:10.3389/fnut.2026.1881228)
Supplement: Supplementary file 1 [file Data_Sheet_1.ZIP › Supplementary Table S4.docx]

**Supplementary Table S4.** Derivation robustness checks, repeat-measurement stability metrics, and first-to-last examination gap distribution.

**Panel A.** Robustness of projected scores after exclusion of BMI or WHtR from the input set.

| Sensitivity scenario | PC1 score correlation | PC2 score correlation | PC3 score correlation |
| --- | --- | --- | --- |
| Exclude BMI | 0.9990 | 0.9997 | 0.9987 |
| Exclude WHtR | 0.9980 | 0.9987 | 0.9996 |

**Panel B.** Repeat-measurement stability based on first-versus-last projected scores.

| Repeatability metric | Correlation |
| --- | --- |
| PC1 first-vs-last correlation | 0.8079 |
| PC2 first-vs-last correlation | 0.8501 |
| PC3 first-vs-last correlation | 0.7381 |

**Panel C.** Distribution of time gaps between first and last valid examinations in the repeat-measurement subset.

| First-to-last examination gap | Patients, n |
| --- | --- |
| ≤90 days | 1213 |
| 91–365 days | 1248 |
| >365 days | 652 |
| Total row | 3113 |

**Note:** Panel A reports correlations between the reference projected scores from the six-variable derivation model and the corresponding scores obtained after repeating PCA with exclusion of BMI or WHtR from the input set. Panel B reports first-versus-last correlations for projected PC scores in the repeat-measurement subset, using the frozen derivation-defined phenotype space. Panel C summarizes the distribution of time gaps between the first and last valid examinations in that subset. Together, these results support high robustness to modest perturbation of the input set and moderate-to-high repeatability of the phenotype axes over time.
