# Supplementary material for: Body-composition phenotype axes provide additional explanatory information for cardiorespiratory fitness and liver-related metabolic risk within BMI strata
Source: Front Nutr. 2026 Aug 31;13:1881228. doi: 10.3389/fnut.2026.1881228 (PMC13572601; doi:10.3389/fnut.2026.1881228)
Supplement: Supplementary file 1 [file Data_Sheet_1.ZIP › Supplementary Table S5.docx]

**Supplementary Table S5.** Age-residualization anchors and knot-sensitivity diagnostics for external projection

Panel A1. Derivation-defined residualization anchors used for external projection

| Item | Value | Interpretation |
| --- | --- | --- |
| Source cohort | Outpatient derivation cohort | Age-residualization specification locked from the original PCA derivation step |
| Participants contributing to PCA derivation, n | 23,698 | First valid examination per participant |
| Age, mean ± SD, years | 40.2 ± 16.1 | Reported in the manuscript and Table 1 |
| Age term in residualization model | Natural spline of age (df = 3) | Applied to each body-composition input before PCA and projection |
| Internal knots | 31 and 46 years | Derivation-defined knots retained unchanged for NHANES projection |
| Knot derivation | 33rd and 67th percentiles of age in the derivation cohort | Defines the original residualization basis |
| Interaction term | Age-by-sex interaction | Model formula: Y ~ ns(Age, df = 3) * Sex |

Panel A2. NHANES age distribution and coverage of derivation-defined knots

| Sample | n | Weighted summary | Mean ± SD, years | Median, years | NHANES 33rd/67th percentiles, years | <31 y, % | 31–46 y, % | >46 y, % | Interpretation |
| --- | --- | --- | --- | --- | --- | --- | --- | --- | --- |
| NHANES analytic base sample | 5,060 | Yes | 35.0 ± 8.7 | 35 | 30/40 | 33.6 | 56.5 | 9.9 | Primary knots evaluated against observed age range |
| Estimated VO₂max analytic subset | 2,721 | Yes | 33.8 ± 8.6 | 34 | 29/38 | 37.8 | 54.2 | 7.9 | Primary knots evaluated against observed age range |
| ALT analytic subset | 4,816 | Yes | 35.0 ± 8.6 | 36 | 31/40 | 33.2 | 57.1 | 9.8 | Primary knots evaluated against observed age range |

Panel B. Residualization and knot-sensitivity score correlations

| Sensitivity scenario | PC1 r | PC2 r | PC3 r | n compared | Interpretation |
| --- | --- | --- | --- | --- | --- |
| Reconstructed primary residualization using derivation-defined knots 31/46 | 1 | 1 | 1 | 5,055 | High score concordance with the submitted primary projection |
| NHANES-specific internal knots 30.0/40.0 | 1 | 1 | 1 | 5,055 | High score concordance with the submitted primary projection |
| Linear age-by-sex residualization | 0.999 | 0.9954 | 0.9998 | 5,055 | High score concordance with the submitted primary projection |

Panel C. Optional outcome-association directional concordance

| Outcome | Sensitivity scenario | Concordant tests | Total tests | Concordance, % | Minimum stratum n | Interpretation |
| --- | --- | --- | --- | --- | --- | --- |
| ALT | Linear age-by-sex | 12/12 | 12 | 100 | 327 | Direction of outcome associations was highly preserved |
| ALT | NHANES-specific knots | 12/12 | 12 | 100 | 327 | Direction of outcome associations was highly preserved |
| Estimated VO₂max | Linear age-by-sex | 12/12 | 12 | 100 | 195 | Direction of outcome associations was highly preserved |
| Estimated VO₂max | NHANES-specific knots | 12/12 | 12 | 100 | 195 | Direction of outcome associations was highly preserved |

**Notes:** Projected PC scores represent age- and sex-residualized body-composition phenotype scores rather than raw body-composition values. The derivation-defined knots of 31 and 46 years were retained from the original PCA residualization specification and correspond to the 33rd and 67th percentiles of age in the outpatient derivation cohort. Panel A1 reports the locked derivation specification; median age and knot-coverage percentages for the derivation cohort are not shown because the available derivation RDS object is a prcomp object rather than an individual-level data frame. Panel A2 evaluates whether the NHANES 20–49-year external projection samples cover the derivation-defined knots. Panel B compares projected PC scores obtained under alternative age-residualization specifications with the submitted primary projection. High correlations indicate that NHANES projection was not materially dependent on exact knot placement. Panel C is supportive only and should not be interpreted as an additional primary outcome-analysis hierarchy.

Abbreviations: PC, principal component; NHANES, National Health and Nutrition Examination Survey; ALT, alanine aminotransferase; Estimated VO₂max, estimated maximal oxygen uptake.
