# Supplementary material for: Body-composition phenotype axes provide additional explanatory information for cardiorespiratory fitness and liver-related metabolic risk within BMI strata
Source: Front Nutr. 2026 Aug 31;13:1881228. doi: 10.3389/fnut.2026.1881228 (PMC13572601; doi:10.3389/fnut.2026.1881228)
Supplement: Supplementary file 1 [file Data_Sheet_1.ZIP › Supplementary Table S6.docx]

**Supplementary Table S6.** Weighted characteristics of participants with versus without estimated VO_2_max availability in the NHANES analytic base sample.

| Characteristic | Unavailable | Available |
| --- | --- | --- |
| Unweighted n | 2339 | 2721 |
| Weighted population total | 40490834.40 | 50795658.33 |
| Age, years | 36.50 (8.46) | 33.79 (8.61) |
| Male, % | 49.27 | 52.26 |
| Hispanic, % | 16.42 | 15.53 |
| Non-Hispanic White, % | 64.59 | 70.18 |
| Non-Hispanic Black, % | 12.87 | 10.03 |
| Other, % | 6.12 | 4.27 |
| BMI, kg/m² | 27.85 (6.06) | 26.86 (5.54) |
| WHtR | 0.56 (0.09) | 0.54 (0.08) |
| FMI, kg/m² | 9.36 (4.43) | 8.40 (4.07) |
| FFMI, kg/m² | 18.50 (3.69) | 18.46 (3.71) |
| PBF, % | 32.60 (10.50) | 30.48 (10.48) |
| PhA, ° | 6.82 (0.90) | 6.93 (0.87) |
| PC1 | -0.02 (1.02) | -0.16 (0.96) |
| PC2 | -0.12 (1.00) | 0.00 (0.96) |
| PC3 | -0.01 (1.00) | 0.01 (0.96) |

**Note:** Values are presented as weighted means (SDs) or weighted percentages using MEC examination weights, unless otherwise indicated. “Unweighted n” denotes the observed sample size in each subgroup, whereas “Weighted population total” denotes the corresponding weighted population estimate. Participants were grouped according to the availability of estimated VO_2_max in the NHANES analytic base sample. This table was intended to characterize the availability profile of the fitness subset rather than to reproduce the overall analytic-base-sample summary shown in Table 1. WHtR, waist-to-height ratio; FMI, fat mass index; FFMI, fat-free mass index; PBF, percent body fat; PhA, phase angle.
