# Supplementary material for: Body-composition phenotype axes provide additional explanatory information for cardiorespiratory fitness and liver-related metabolic risk within BMI strata
Source: Front Nutr. 2026 Aug 31;13:1881228. doi: 10.3389/fnut.2026.1881228 (PMC13572601; doi:10.3389/fnut.2026.1881228)
Supplement: Supplementary file 1 [file Data_Sheet_1.ZIP › Supplementary Table S7.docx]

**Supplementary Table S7.** Estimated VO₂max protocol-related sensitivity analyses

Panel A. Protocol-variable availability and analysis feasibility

| Item | Result |
| --- | --- |
| NHANES analytic rows in merged dataset | 5060 |
| Estimated VO₂max column | cvdesvo2 |
| Candidate protocol variable | cvaprot |
| Protocol selection note | CVAPROT was used as the protocol-assignment covariate based on the NHANES cardiovascular fitness component documentation. |
| Protocol variable non-missing among estimated VO₂ analytic rows | 2498 |
| Protocol variable levels among estimated VO₂ analytic rows | 4; 3; 5; 6; 1; 2; 8 |
| Protocol variable selection | CVAPROT was used as the protocol-assignment covariate because it was available across all three cycles and identified as the assigned exercise protocol variable. |
| Restricted-protocol exclusion | A restricted-protocol exclusion analysis was not performed because a reproducible public-use flag defining modified-protocol assignment could not be harmonized across the included cycles. |

Panel B. Cross-cycle CVX variables relevant to protocol sensitivity

| Variable | Cycles present | n cycles | Example label | Use in S16 |
| --- | --- | --- | --- | --- |
| CVAPROT | 1999_2000;2001_2002;2003_2004 | 3 | Assigned exercise protocol | Selected protocol-assignment covariate |
| CVDPROT | 1999_2000;2001_2002;2003_2004 | 3 | Exercise protocol used in stage 1 and 2 | Secondary protocol variable; not selected as primary protocol covariate |
| CVDESVO2 | 1999_2000;2001_2002;2003_2004 | 3 | Estimated VO₂max (ml/kg/min) | Estimated VO₂max outcome |
| CVDEXLEN | 1999_2000;2001_2002;2003_2004 | 3 | Length of CV fitness exam (min) | Fitness exam length diagnostic |
| CVDEXSTS | 1999_2000;2001_2002;2003_2004 | 3 | CV fitness exam status | Fitness exam status diagnostic |
| CVDFITLV | 1999_2000;2001_2002;2003_2004 | 3 | Cardiovascular fitness level | Cardiovascular fitness level diagnostic |

Panel C. Estimated VO₂max protocol-related sensitivity models

| Model | BMI quartile | n | Phenotype axis | β coefficient (95% CI) | p value |
| --- | --- | --- | --- | --- | --- |
| Primary demographic model | Q1 | 685 | PC1 | −3.48 (−6.38 to −0.57) | 0.02 |
| Primary demographic model | Q1 | 685 | PC2 | 1.46 (0.58 to 2.33) | 0.002 |
| Primary demographic model | Q1 | 685 | PC3 | 1.06 (−0.08 to 2.20) | 0.068 |
| Primary demographic model | Q2 | 675 | PC1 | −1.52 (−4.77 to 1.74) | 0.35 |
| Primary demographic model | Q2 | 675 | PC2 | 0.44 (−0.44 to 1.33) | 0.314 |
| Primary demographic model | Q2 | 675 | PC3 | 1.37 (0.11 to 2.63) | 0.033 |
| Primary demographic model | Q3 | 724 | PC1 | −2.81 (−5.93 to 0.31) | 0.076 |
| Primary demographic model | Q3 | 724 | PC2 | 1.82 (0.36 to 3.27) | 0.016 |
| Primary demographic model | Q3 | 724 | PC3 | 0.35 (−0.62 to 1.32) | 0.465 |
| Primary demographic model | Q4 | 637 | PC1 | −2.64 (−4.62 to −0.67) | 0.01 |
| Primary demographic model | Q4 | 637 | PC2 | 0.58 (−0.17 to 1.33) | 0.127 |
| Primary demographic model | Q4 | 637 | PC3 | 1.72 (0.58 to 2.87) | 0.004 |
| Expanded covariate model | Q1 | 660 | PC1 | −3.42 (−6.43 to −0.41) | 0.027 |
| Expanded covariate model | Q1 | 660 | PC2 | 1.21 (0.32 to 2.11) | 0.01 |
| Expanded covariate model | Q1 | 660 | PC3 | 1.11 (−0.06 to 2.27) | 0.061 |
| Expanded covariate model | Q2 | 644 | PC1 | −2.16 (−5.86 to 1.53) | 0.241 |
| Expanded covariate model | Q2 | 644 | PC2 | 0.42 (−0.53 to 1.36) | 0.375 |
| Expanded covariate model | Q2 | 644 | PC3 | 1.27 (−0.05 to 2.59) | 0.06 |
| Expanded covariate model | Q3 | 696 | PC1 | −2.87 (−5.90 to 0.16) | 0.063 |
| Expanded covariate model | Q3 | 696 | PC2 | 1.79 (0.33 to 3.25) | 0.018 |
| Expanded covariate model | Q3 | 696 | PC3 | 0.61 (−0.40 to 1.62) | 0.224 |
| Expanded covariate model | Q4 | 614 | PC1 | −2.71 (−4.66 to −0.75) | 0.009 |
| Expanded covariate model | Q4 | 614 | PC2 | 0.91 (0.10 to 1.72) | 0.029 |
| Expanded covariate model | Q4 | 614 | PC3 | 1.60 (0.44 to 2.75) | 0.008 |
| Protocol-adjusted demographic model | Q1 | 574 | PC1 | −2.78 (−5.51 to −0.06) | 0.045 |
| Protocol-adjusted demographic model | Q1 | 574 | PC2 | 1.17 (0.15 to 2.18) | 0.026 |
| Protocol-adjusted demographic model | Q1 | 574 | PC3 | 0.90 (−0.43 to 2.23) | 0.179 |
| Protocol-adjusted demographic model | Q2 | 608 | PC1 | −0.54 (−4.61 to 3.53) | 0.789 |
| Protocol-adjusted demographic model | Q2 | 608 | PC2 | 0.43 (−0.61 to 1.46) | 0.408 |
| Protocol-adjusted demographic model | Q2 | 608 | PC3 | 1.30 (0.04 to 2.56) | 0.043 |
| Protocol-adjusted demographic model | Q3 | 682 | PC1 | −2.12 (−5.15 to 0.91) | 0.164 |
| Protocol-adjusted demographic model | Q3 | 682 | PC2 | 1.11 (0.24 to 1.98) | 0.014 |
| Protocol-adjusted demographic model | Q3 | 682 | PC3 | 0.48 (−0.47 to 1.44) | 0.311 |
| Protocol-adjusted demographic model | Q4 | 634 | PC1 | −3.61 (−5.77 to −1.45) | 0.002 |
| Protocol-adjusted demographic model | Q4 | 634 | PC2 | 0.74 (−0.06 to 1.54) | 0.068 |
| Protocol-adjusted demographic model | Q4 | 634 | PC3 | 1.58 (0.36 to 2.81) | 0.013 |
| Expanded + protocol-adjusted model | Q1 | 554 | PC1 | −2.85 (−5.81 to 0.11) | 0.058 |
| Expanded + protocol-adjusted model | Q1 | 554 | PC2 | 1.03 (−0.00 to 2.05) | 0.05 |
| Expanded + protocol-adjusted model | Q1 | 554 | PC3 | 0.94 (−0.34 to 2.22) | 0.141 |
| Expanded + protocol-adjusted model | Q2 | 577 | PC1 | −0.73 (−5.34 to 3.89) | 0.747 |
| Expanded + protocol-adjusted model | Q2 | 577 | PC2 | 0.36 (−0.71 to 1.44) | 0.492 |
| Expanded + protocol-adjusted model | Q2 | 577 | PC3 | 1.18 (−0.15 to 2.52) | 0.08 |
| Expanded + protocol-adjusted model | Q3 | 655 | PC1 | −2.33 (−5.17 to 0.50) | 0.102 |
| Expanded + protocol-adjusted model | Q3 | 655 | PC2 | 1.14 (0.20 to 2.08) | 0.02 |
| Expanded + protocol-adjusted model | Q3 | 655 | PC3 | 0.66 (−0.38 to 1.70) | 0.202 |
| Expanded + protocol-adjusted model | Q4 | 611 | PC1 | −3.57 (−5.75 to −1.39) | 0.002 |
| Expanded + protocol-adjusted model | Q4 | 611 | PC2 | 1.03 (0.18 to 1.87) | 0.019 |
| Expanded + protocol-adjusted model | Q4 | 611 | PC3 | 1.46 (0.25 to 2.67) | 0.02 |

**Note:**

Values in Panel C are survey-weighted β coefficients with 95% confidence intervals per 1-SD increment in the projected phenotype axis.

The outcome was estimated VO₂max from the NHANES cardiovascular fitness examination. Primary demographic models were adjusted for age, sex, and race/ethnicity. Expanded covariate models additionally included physical activity, smoking status, and recent health status. Protocol-adjusted models additionally included the treadmill protocol-assignment variable CVAPROT. CVAPROT was selected because the NHANES CVX variable documentation identifies it as the assigned exercise protocol variable and it was present across all three NHANES cycles used in this analysis. CVDPROT was retained as a secondary protocol-related diagnostic variable. A restricted-protocol exclusion analysis was not fitted because a reproducible public-use modified-protocol flag/value set was not available from the current outputs. Estimated VO₂max should therefore be interpreted as an estimated, protocol-derived functional endpoint rather than directly measured cardiorespiratory fitness. BMI quartiles were the weighted unified BMI quartiles defined from the NHANES analytic base sample and applied consistently across outcome analyses.

Abbreviations: BMI, body mass index; CI, confidence interval; CVX, cardiovascular fitness component; NHANES, National Health and Nutrition Examination Survey; PC, principal component; Estimated VO₂max, estimated maximal oxygen uptake.
