# Supplementary material for: Body-composition phenotype axes provide additional explanatory information for cardiorespiratory fitness and liver-related metabolic risk within BMI strata
Source: Front Nutr. 2026 Aug 31;13:1881228. doi: 10.3389/fnut.2026.1881228 (PMC13572601; doi:10.3389/fnut.2026.1881228)
Supplement: Supplementary file 1 [file Data_Sheet_1.ZIP › Supplementary Table S8.docx]

**Supplementary Table S8.** ALT clean-sample sensitivity analyses excluding heavy alcohol use and positive hepatitis serology

Panel A. Primary clean-sample model

| BMI quartile | n | Phenotype axis | ALT percent difference (95% CI) | p value |
| --- | --- | --- | --- | --- |
| Q1 | 802 | PC1 | 19.45% (6.31% to 34.22%) | 0.004 |
| Q1 | 802 | PC2 | 2.22% (−2.52% to 7.18%) | 0.354 |
| Q1 | 802 | PC3 | −5.49% (−11.00% to 0.36%) | 0.064 |
| Q2 | 849 | PC1 | 7.55% (−7.31% to 24.79%) | 0.327 |
| Q2 | 849 | PC2 | 3.97% (0.27% to 7.81%) | 0.036 |
| Q2 | 849 | PC3 | −1.97% (−7.48% to 3.87%) | 0.49 |
| Q3 | 892 | PC1 | 23.35% (5.80% to 43.82%) | 0.009 |
| Q3 | 892 | PC2 | 2.35% (−1.37% to 6.20%) | 0.212 |
| Q3 | 892 | PC3 | −5.10% (−9.16% to −0.86%) | 0.02 |
| Q4 | 979 | PC1 | 16.41% (9.67% to 23.56%) | <0.001 |
| Q4 | 979 | PC2 | 7.59% (3.64% to 11.68%) | <0.001 |
| Q4 | 979 | PC3 | −5.57% (−10.48% to −0.38%) | 0.036 |

Panel B. Expanded clean-sample model

| BMI quartile | n | Phenotype axis | ALT percent difference (95% CI) | p value |
| --- | --- | --- | --- | --- |
| Q1 | 534 | PC1 | 18.27% (0.88% to 38.66%) | 0.039 |
| Q1 | 534 | PC2 | 0.13% (−5.05% to 5.59%) | 0.96 |
| Q1 | 534 | PC3 | −2.59% (−7.99% to 3.12%) | 0.352 |
| Q2 | 554 | PC1 | 7.06% (−11.26% to 29.17%) | 0.462 |
| Q2 | 554 | PC2 | 3.30% (−0.14% to 6.87%) | 0.06 |
| Q2 | 554 | PC3 | −1.02% (−6.88% to 5.22%) | 0.734 |
| Q3 | 541 | PC1 | 25.07% (5.28% to 48.58%) | 0.013 |
| Q3 | 541 | PC2 | 5.19% (0.41% to 10.20%) | 0.034 |
| Q3 | 541 | PC3 | −6.18% (−12.01% to 0.04%) | 0.051 |
| Q4 | 534 | PC1 | 14.52% (4.77% to 25.17%) | 0.004 |
| Q4 | 534 | PC2 | 7.34% (2.39% to 12.53%) | 0.005 |
| Q4 | 534 | PC3 | −3.28% (−10.56% to 4.60%) | 0.389 |

**Note:**

The ALT clean sample excluded participants with heavy alcohol use and participants with positive hepatitis serology. Models were fitted separately within the primary weighted unified BMI quartiles. ALT was modeled on the natural-log scale and back-transformed; effect estimates are shown as percent differences with 95% confidence intervals per 1-SD increment in the corresponding projected phenotype axis. The primary clean-sample model adjusted for age, sex, and race/ethnicity. The expanded clean-sample model additionally adjusted for physical activity, alcohol intake, smoking status, and diabetes status. These analyses were conducted as sensitivity analyses for the ALT endpoint and should be interpreted as supportive rather than as a separate primary evidentiary hierarchy.

Abbreviations: ALT, alanine aminotransferase; BMI, body mass index; CI, confidence interval; PC, principal component; SD, standard deviation.
