# Supplementary material for: Body-composition phenotype axes provide additional explanatory information for cardiorespiratory fitness and liver-related metabolic risk within BMI strata
Source: Front Nutr. 2026 Aug 31;13:1881228. doi: 10.3389/fnut.2026.1881228 (PMC13572601; doi:10.3389/fnut.2026.1881228)
Supplement: Supplementary file 1 [file Data_Sheet_1.ZIP › Supplementary Table S9.docx]

**Supplementary Table S9.** Complete-case comparison for primary and expanded covariate-adjusted NHANES sensitivity models

| Outcome | Analytic subset | n | Weighted population total, millions | Age, years | BMI, kg/m² | PC1 mean | PC2 mean | PC3 mean | Male, % | Current smoking, % | Diabetes, % | Heavy alcohol use, % | Positive hepatitis serology, % |
| --- | --- | --- | --- | --- | --- | --- | --- | --- | --- | --- | --- | --- | --- |
| Estimated VO₂max | Primary complete-case subset | 2721 | 50.8 | 33.8 ± 8.6 | 26.9 ± 5.5 | −0.155 | −0.003 | 0.011 | 52.3 | 26.7 | 1.2 | 32.9 | 1 |
| Estimated VO₂max | Expanded complete-case subset | 2614 | 48.91 | 33.9 ± 8.6 | 26.9 ± 5.6 | −0.154 | 0 | 0.013 | 52.4 | 26.8 | 1.2 | 32.9 | 1 |
| ALT | Primary complete-case subset | 4857 | 88.02 | 35.0 ± 8.6 | 27.3 ± 5.8 | −0.092 | −0.048 | 0.001 | 51 | 29.5 | 2.6 | 33.8 | 2.1 |
| ALT | Expanded complete-case subset | 3455 | 65.06 | 35.0 ± 8.5 | 27.0 ± 5.5 | −0.143 | −0.028 | −0.023 | 53.6 | 32.5 | 2.2 | 33.7 | 1.9 |

**Notes:**

Values are weighted estimates unless otherwise indicated; n denotes the unweighted sample size.

The primary complete-case subset includes participants with non-missing projected phenotype axes, the corresponding outcome, positive MEC examination weights, and complete demographic covariates for the original age-, sex-, and race/ethnicity-adjusted model.

The expanded complete-case subset additionally required complete reviewer-requested behavioral and clinical covariates. For estimated VO₂max, the expanded model additionally included physical activity, smoking status, and recent health status. For ALT, the expanded model additionally included physical activity, alcohol intake, smoking status, and diabetes status.

Weighted population totals are shown in millions of persons. Age and BMI are presented as weighted mean ± SD. PC1–PC3 values are weighted means of projected phenotype scores.

Heavy alcohol use was defined operationally for sensitivity analysis. Positive hepatitis serology indicates evidence of positive hepatitis-related serologic markers used to define the ALT clean-sample sensitivity analysis.

Abbreviations: BMI, body mass index; ALT, alanine aminotransferase; PC, principal component; SD, standard deviation; NHANES, National Health and Nutrition Examination Survey.
