# Supplementary material for: Body-composition phenotype axes provide additional explanatory information for cardiorespiratory fitness and liver-related metabolic risk within BMI strata
Source: Front Nutr. 2026 Aug 31;13:1881228. doi: 10.3389/fnut.2026.1881228 (PMC13572601; doi:10.3389/fnut.2026.1881228)
Supplement: Supplementary file 1 [file Data_Sheet_1.ZIP › Supplementary Table S10.docx]

**Supplementary Table S10.** Covariate harmonization summary for expanded and clean-sample sensitivity analyses

| Domain | Harmonized variables | Use in revised analyses |
| --- | --- | --- |
| Physical activity | pa_level, pa_any_vigmod, pa_vigorous30, pa_moderate30, pa_strength30 | estimated VO₂max and ALT expanded adjustment; pa_any_vigmod used to limit missingness |
| Alcohol | alcohol_drinks_per_day, heavy_alcohol_operational | ALT expanded adjustment; heavy-alcohol exclusion for ALT clean-sample sensitivity |
| Smoking | smoking_status, cigs_per_day_now | estimated VO₂max and ALT expanded adjustment |
| Diabetes | diabetes_status, insulin_now, diabetes_pills | ALT expanded adjustment as metabolic-status covariate |
| Hepatitis serology | hep_any_serology_pos, hbv_surface_antigen_pos, hcv_confirmed_ab_pos, hcv_rna_pos | ALT clean-sample sensitivity exclusion |
| Health status | recent_health_4w, general_health_2cycle | estimated VO₂max expanded adjustment; general_health_2cycle retained as secondary only |
| Prescription medication | any_rx_use, rx_count | Descriptive/supportive only; not used for hepatotoxic-medication exclusion because drug-level hepatotoxicity cannot be reliably harmonized here |

**Note:**

This table summarizes reviewer-requested covariate domains harmonized across NHANES 1999–2004 for the expanded covariate and clean-sample sensitivity analyses. Physical activity, smoking status, and recent health status were used for estimated VO₂max expanded adjustment. Physical activity, alcohol intake, smoking status, and diabetes status were used for ALT expanded adjustment. The ALT clean-sample sensitivity analysis excluded participants with heavy alcohol use and positive hepatitis serology. Prescription medication variables were retained only for descriptive/supportive assessment; drug-specific hepatotoxic medication exclusion was not implemented because reliable cross-cycle harmonization of hepatotoxic drug classes was not feasible from the available public-use variables.

Abbreviations: ALT, alanine aminotransferase; NHANES, National Health and Nutrition Examination Survey.
