# Supplementary material for: Body-composition phenotype axes provide additional explanatory information for cardiorespiratory fitness and liver-related metabolic risk within BMI strata
Source: Front Nutr. 2026 Aug 31;13:1881228. doi: 10.3389/fnut.2026.1881228 (PMC13572601; doi:10.3389/fnut.2026.1881228)
Supplement: Supplementary file 1 [file Data_Sheet_1.ZIP › Supplementary Table S11.docx]

**Supplementary Table S11.** Sensitivity analyses of associations of projected phenotype axes with estimated VO_2_max and ALT across WHO BMI categories (<25, 25–29.9, and ≥30 kg/m²).

**Panel A.** Estimated VO_2_max: survey-weighted associations across WHO BMI categories.

| WHO BMI category | n | PC1 effect (95% CI) | PC1 p | PC2 effect (95% CI) | PC2 p | PC3 effect (95% CI) | PC3 p |
| --- | --- | --- | --- | --- | --- | --- | --- |
| <25 kg/m² | 1092 | -3.50 (-5.47 to -1.53) | 0.001 | 1.19 (0.52 to 1.85) | 0.001 | 1.24 (0.43 to 2.05) | 0.005 |
| 25–29.9 kg/m² | 922 | -3.03 (-5.19 to -0.87) | 0.009 | 1.46 (0.46 to 2.46) | 0.007 | 0.25 (-0.70 to 1.20) | 0.606 |
| ≥30 kg/m² | 707 | -2.04 (-3.88 to -0.19) | 0.037 | 0.61 (-0.07 to 1.29) | 0.089 | 1.58 (0.47 to 2.70) | 0.009 |

**Panel B.** ALT: survey-weighted associations across WHO BMI categories.

| WHO BMI category | n | PC1 effect (95% CI) | PC1 p | PC2 effect (95% CI) | PC2 p | PC3 effect (95% CI) | PC3 p |
| --- | --- | --- | --- | --- | --- | --- | --- |
| <25 kg/m² | 1785 | 17.42% (10.31% to 24.99%) | <0.001 | 1.28% (-1.18% to 3.81%) | 0.317 | -3.55% (-7.45% to 0.51%) | 0.094 |
| 25–29.9 kg/m² | 1641 | 26.00% (14.77% to 38.33%) | <0.001 | 2.51% (-0.78% to 5.90%) | 0.145 | -3.12% (-6.47% to 0.35%) | 0.086 |
| ≥30 kg/m² | 1431 | 16.90% (10.87% to 23.26%) | <0.001 | 6.62% (3.22% to 10.13%) | <0.001 | -6.09% (-9.83% to -2.19%) | 0.005 |

**Note:** Survey-weighted regression models were fitted separately within WHO BMI categories (<25, 25–29.9, and ≥30 kg/m²) as a sensitivity analysis complementary to the primary weighted unified BMI quartile analysis. Models were adjusted for age, sex, and race/ethnicity. For estimated VO_2_max, effects are shown as adjusted β coefficients with 95% confidence intervals per 1-SD increment in the corresponding projected phenotype axis. For ALT, models were fitted on the natural-log scale and back-transformed; effects are therefore shown as percent differences with 95% confidence intervals per 1-SD increment in the corresponding projected phenotype axis. Projected phenotype axes were derived by external projection of NHANES data into the derivation-defined phenotype space.

ALT, alanine aminotransferase.
