# Supplementary material for: Body-composition phenotype axes provide additional explanatory information for cardiorespiratory fitness and liver-related metabolic risk within BMI strata
Source: Front Nutr. 2026 Aug 31;13:1881228. doi: 10.3389/fnut.2026.1881228 (PMC13572601; doi:10.3389/fnut.2026.1881228)
Supplement: Supplementary file 1 [file Data_Sheet_1.ZIP › Supplementary Table S12.docx]

**Supplementary Table S12.** Sensitivity analyses of associations of projected phenotype axes with estimated VO_2_max and ALT using outcome-specific BMI quartiles, including comparison of weighted unified and outcome-specific BMI quartile cutpoints.

**Panel A.** Comparison of weighted unified and outcome-specific BMI quartile cutpoints.

| Method | Q1 cutoff (kg/m²) | Q2 cutoff (kg/m²) | Q3 cutoff (kg/m²) | Δ from unified Q1 | Δ from unified Q2 | Δ from unified Q3 |
| --- | --- | --- | --- | --- | --- | --- |
| Unified (Analytic Sample) | 23.16 | 26.37 | 30.45 | 0.00 | 0.00 | 0.00 |
| VO_2_-specific | 22.86 | 25.96 | 29.77 | -0.30 | -0.41 | -0.68 |
| ALT-specific | 23.21 | 26.40 | 30.41 | 0.05 | 0.03 | -0.04 |

**Panel B.** Estimated VO_2_max: survey-weighted associations using outcome-specific BMI quartiles.

| Outcome-specific BMI quartile | n | PC1 effect (95% CI) | PC1 p | PC2 effect (95% CI) | PC2 p | PC3 effect (95% CI) | PC3 p |
| --- | --- | --- | --- | --- | --- | --- | --- |
| Q1 | 622 | -3.10 (-6.12 to -0.09) | 0.051 | 1.29 (0.41 to 2.16) | 0.007 | 1.14 (0.08 to 2.20) | 0.042 |
| Q2 | 651 | -1.92 (-5.29 to 1.45) | 0.272 | 0.72 (-0.19 to 1.63) | 0.129 | 1.30 (0.20 to 2.40) | 0.026 |
| Q3 | 701 | -2.70 (-5.32 to -0.09) | 0.050 | 1.76 (0.50 to 3.02) | 0.010 | 0.39 (-0.63 to 1.41) | 0.461 |
| Q4 | 747 | -1.96 (-3.71 to -0.20) | 0.035 | 0.55 (-0.18 to 1.29) | 0.148 | 1.64 (0.54 to 2.75) | 0.006 |

**Panel C.** ALT: survey-weighted associations using outcome-specific BMI quartiles.

| Outcome-specific BMI quartile | n | PC1 effect (95% CI) | PC1 p | PC2 effect (95% CI) | PC2 p | PC3 effect (95% CI) | PC3 p |
| --- | --- | --- | --- | --- | --- | --- | --- |
| Q1 | 1119 | 19.77% (8.64% to 32.03%) | <0.001 | 1.41% (-2.29% to 5.26%) | 0.465 | -2.66% (-8.13% to 3.15%) | 0.368 |
| Q2 | 1170 | 17.36% (4.07% to 32.35%) | 0.013 | 1.82% (-1.80% to 5.57%) | 0.336 | -3.18% (-8.01% to 1.90%) | 0.223 |
| Q3 | 1240 | 15.45% (1.28% to 31.61%) | 0.038 | 4.32% (1.21% to 7.53%) | 0.009 | -5.44% (-8.73% to -2.02%) | 0.004 |
| Q4 | 1328 | 17.28% (11.48% to 23.37%) | <0.001 | 6.75% (3.16% to 10.47%) | <0.001 | -6.54% (-10.28% to -2.64%) | 0.003 |

**Note:** Panel A compares the primary weighted unified BMI quartile cutpoints with the outcome-specific BMI quartile cutpoints obtained separately for the estimated VO_2_max and ALT analytic subsets. Panels B and C report survey-weighted regression models fitted within those outcome-specific BMI quartiles as a sensitivity analysis complementary to the primary weighted unified BMI quartile framework. Models were adjusted for age, sex, and race/ethnicity. For estimated VO_2_max, effects are shown as adjusted β coefficients with 95% confidence intervals per 1-SD increment in the corresponding projected phenotype axis. For ALT, models were fitted on the natural-log scale and back-transformed; effects are therefore shown as percent differences with 95% confidence intervals per 1-SD increment in the corresponding projected phenotype axis. ALT, alanine aminotransferase.
