# Supplementary material for: Body-composition phenotype axes provide additional explanatory information for cardiorespiratory fitness and liver-related metabolic risk within BMI strata
Source: Front Nutr. 2026 Aug 31;13:1881228. doi: 10.3389/fnut.2026.1881228 (PMC13572601; doi:10.3389/fnut.2026.1881228)
Supplement: Supplementary file 1 [file Data_Sheet_1.ZIP › Supplementary Table S13.docx]

**Supplementary Table S13.** Sequentially adjusted models for associations of projected phenotype axes with estimated VO_2_max and ALT within weighted unified BMI quartiles.

**Panel A.** Estimated VO_2_max: sequentially adjusted survey-weighted models within weighted unified BMI quartiles.

| BMI quartile | Model | n | PC1 effect (95% CI) | PC1 p | PC2 effect (95% CI) | PC2 p | PC3 effect (95% CI) | PC3 p |
| --- | --- | --- | --- | --- | --- | --- | --- | --- |
| Q1 | Model 0 | 685 | 2.58 (-0.08 to 5.24) | 0.065 | 0.74 (-0.14 to 1.62) | 0.105 | 0.97 (-0.19 to 2.13) | 0.108 |
| Q1 | Model 1 | 685 | -3.30 (-5.98 to -0.61) | 0.021 | 1.44 (0.58 to 2.30) | 0.002 | 1.21 (0.13 to 2.29) | 0.034 |
| Q1 | Model 2 | 685 | -3.48 (-6.28 to -0.67) | 0.021 | 1.46 (0.61 to 2.30) | 0.002 | 1.06 (-0.04 to 2.16) | 0.068 |
| Q2 | Model 0 | 675 | 5.71 (2.84 to 8.57) | <0.001 | -0.35 (-1.21 to 0.51) | 0.428 | 2.95 (1.65 to 4.26) | <0.001 |
| Q2 | Model 1 | 675 | -2.07 (-5.14 to 1.00) | 0.194 | 0.52 (-0.36 to 1.40) | 0.254 | 1.32 (0.11 to 2.53) | 0.040 |
| Q2 | Model 2 | 675 | -1.52 (-4.66 to 1.62) | 0.350 | 0.44 (-0.41 to 1.30) | 0.314 | 1.37 (0.16 to 2.59) | 0.034 |
| Q3 | Model 0 | 724 | 2.91 (0.01 to 5.81) | 0.056 | 0.82 (-0.44 to 2.09) | 0.210 | 2.22 (0.94 to 3.49) | 0.001 |
| Q3 | Model 1 | 724 | -2.72 (-5.81 to 0.36) | 0.092 | 1.74 (0.44 to 3.05) | 0.013 | 0.48 (-0.44 to 1.41) | 0.310 |
| Q3 | Model 2 | 724 | -2.81 (-5.83 to 0.20) | 0.076 | 1.82 (0.41 to 3.22) | 0.016 | 0.35 (-0.58 to 1.29) | 0.465 |
| Q4 | Model 0 | 637 | -3.77 (-5.80 to -1.74) | <0.001 | 0.13 (-0.62 to 0.87) | 0.743 | 2.48 (1.34 to 3.62) | <0.001 |
| Q4 | Model 1 | 637 | -2.84 (-4.82 to -0.86) | 0.008 | 0.39 (-0.34 to 1.11) | 0.305 | 1.83 (0.70 to 2.95) | 0.003 |
| Q4 | Model 2 | 637 | -2.64 (-4.56 to -0.73) | 0.010 | 0.58 (-0.15 to 1.30) | 0.127 | 1.72 (0.61 to 2.83) | 0.004 |

**Panel B.** ALT: sequentially adjusted survey-weighted models within weighted unified BMI quartiles.

| BMI quartile | Model | n | PC1 effect (95% CI) | PC1 p | PC2 effect (95% CI) | PC2 p | PC3 effect (95% CI) | PC3 p |
| --- | --- | --- | --- | --- | --- | --- | --- | --- |
| Q1 | Model 0 | 1102 | 18.77% (8.86% to 29.58%) | <0.001 | 1.61% (-2.73% to 6.14%) | 0.478 | -7.25% (-12.46% to -1.74%) | 0.014 |
| Q1 | Model 1 | 1102 | 19.06% (8.08% to 31.14%) | 0.001 | 1.51% (-2.16% to 5.33%) | 0.430 | -3.13% (-8.33% to 2.36%) | 0.265 |
| Q1 | Model 2 | 1102 | 19.08% (7.93% to 31.37%) | 0.001 | 1.80% (-2.05% to 5.80%) | 0.370 | -3.57% (-9.06% to 2.25%) | 0.232 |
| Q2 | Model 0 | 1177 | 32.63% (20.62% to 45.84%) | <0.001 | -1.09% (-4.39% to 2.32%) | 0.529 | -0.27% (-5.18% to 4.90%) | 0.917 |
| Q2 | Model 1 | 1177 | 19.18% (6.09% to 33.89%) | 0.005 | 1.29% (-2.22% to 4.94%) | 0.480 | -2.45% (-7.57% to 2.95%) | 0.372 |
| Q2 | Model 2 | 1177 | 15.62% (2.56% to 30.35%) | 0.023 | 1.67% (-1.94% to 5.41%) | 0.376 | -2.74% (-7.72% to 2.50%) | 0.306 |
| Q3 | Model 0 | 1260 | 35.08% (22.24% to 49.27%) | <0.001 | 1.10% (-2.30% to 4.63%) | 0.534 | 0.91% (-2.71% to 4.67%) | 0.628 |
| Q3 | Model 1 | 1260 | 18.38% (3.78% to 35.05%) | 0.016 | 3.54% (0.45% to 6.73%) | 0.030 | -4.61% (-8.21% to -0.88%) | 0.021 |
| Q3 | Model 2 | 1260 | 17.21% (3.05% to 33.31%) | 0.021 | 4.06% (0.91% to 7.31%) | 0.016 | -5.18% (-8.49% to -1.75%) | 0.006 |
| Q4 | Model 0 | 1318 | 5.57% (0.04% to 11.40%) | 0.055 | 4.23% (0.27% to 8.35%) | 0.043 | -0.84% (-5.31% to 3.83%) | 0.721 |
| Q4 | Model 1 | 1318 | 15.50% (10.06% to 21.20%) | <0.001 | 5.78% (2.15% to 9.54%) | 0.003 | -5.70% (-9.43% to -1.81%) | 0.007 |
| Q4 | Model 2 | 1318 | 17.40% (11.61% to 23.49%) | <0.001 | 6.74% (3.23% to 10.38%) | <0.001 | -6.41% (-10.15% to -2.52%) | 0.003 |

**Note:** Sequential models were fitted within the primary weighted unified BMI quartiles. Model 0 included projected phenotype axes only. Model 1 additionally adjusted for age and sex. Model 2 additionally adjusted for age, sex, and race/ethnicity. For estimated VO_2_max, effects are shown as adjusted β coefficients with 95% confidence intervals per 1-SD increment in the corresponding projected phenotype axis. For ALT, models were fitted on the natural-log scale and back-transformed; effects are therefore shown as percent differences with 95% confidence intervals per 1-SD increment in the corresponding projected phenotype axis. Weighted unified BMI quartiles were defined once from the NHANES analytic base sample using MEC examination weights and then applied consistently across all primary models. ALT, alanine aminotransferase.
