# Supplementary material for: Body-composition phenotype axes provide additional explanatory information for cardiorespiratory fitness and liver-related metabolic risk within BMI strata
Source: Front Nutr. 2026 Aug 31;13:1881228. doi: 10.3389/fnut.2026.1881228 (PMC13572601; doi:10.3389/fnut.2026.1881228)
Supplement: Supplementary file 1 [file Data_Sheet_1.ZIP › Supplementary Table S14.docx]

**Supplementary Table S14.** Expanded covariate-adjusted associations of projected phenotype axes with estimated VO_2_max and ALT within unified BMI quartiles

Panel A. Estimated VO_2_max: beta coefficient per 1-SD increment in projected phenotype axis

| BMI quartile | n | Phenotype axis | Effect estimate (95% CI) | p value |
| --- | --- | --- | --- | --- |
| Q1 | 660 | PC1 | −3.42 (−6.43 to −0.41) | 0.027 |
| Q1 | 660 | PC2 | 1.21 (0.32 to 2.11) | 0.01 |
| Q1 | 660 | PC3 | 1.11 (−0.06 to 2.27) | 0.061 |
| Q2 | 644 | PC1 | −2.16 (−5.86 to 1.53) | 0.241 |
| Q2 | 644 | PC2 | 0.42 (−0.53 to 1.36) | 0.375 |
| Q2 | 644 | PC3 | 1.27 (−0.05 to 2.59) | 0.06 |
| Q3 | 696 | PC1 | −2.87 (−5.90 to 0.16) | 0.063 |
| Q3 | 696 | PC2 | 1.79 (0.33 to 3.25) | 0.018 |
| Q3 | 696 | PC3 | 0.61 (−0.40 to 1.62) | 0.224 |
| Q4 | 614 | PC1 | −2.71 (−4.66 to −0.75) | 0.009 |
| Q4 | 614 | PC2 | 0.91 (0.10 to 1.72) | 0.029 |
| Q4 | 614 | PC3 | 1.60 (0.44 to 2.75) | 0.008 |

Panel B. ALT: percent difference per 1-SD increment in projected phenotype axis

| BMI quartile | n | Phenotype axis | Effect estimate (95% CI) | p value |
| --- | --- | --- | --- | --- |
| Q1 | 823 | PC1 | 19.13% (6.27% to 33.56%) | 0.004 |
| Q1 | 823 | PC2 | 0.25% (−3.92% to 4.61%) | 0.905 |
| Q1 | 823 | PC3 | −1.42% (−7.50% to 5.05%) | 0.648 |
| Q2 | 869 | PC1 | 15.49% (−2.69% to 37.06%) | 0.096 |
| Q2 | 869 | PC2 | 1.24% (−1.57% to 4.14%) | 0.377 |
| Q2 | 869 | PC3 | −3.69% (−8.65% to 1.53%) | 0.155 |
| Q3 | 900 | PC1 | 14.66% (−1.36% to 33.28%) | 0.073 |
| Q3 | 900 | PC2 | 6.23% (1.87% to 10.77%) | 0.006 |
| Q3 | 900 | PC3 | −6.51% (−11.22% to −1.55%) | 0.013 |
| Q4 | 863 | PC1 | 16.09% (7.91% to 24.88%) | <0.001 |
| Q4 | 863 | PC2 | 6.49% (2.60% to 10.53%) | 0.002 |
| Q4 | 863 | PC3 | −6.10% (−10.98% to −0.94%) | 0.023 |

**Notes:**

Values are estimates from survey-weighted regression models fitted within unified BMI quartiles. Effects are presented per 1-SD increment in the corresponding projected phenotype axis.

For estimated VO_2_max, models were fitted on the original scale and estimates are shown as beta coefficients with 95% confidence intervals.

For ALT, models were fitted on the natural-log scale and back-transformed; estimates are shown as percent differences in ALT with 95% confidence intervals.

Unified BMI quartiles were defined from the NHANES analytic base sample using MEC examination weights and applied consistently across outcome analyses: Q1, BMI ≤ 23.16 kg/m²; Q2, 23.16 < BMI ≤ 26.37 kg/m²; Q3, 26.37 < BMI ≤ 30.45 kg/m²; Q4, BMI > 30.45 kg/m².

Estimated VO_2_max expanded models were adjusted for age, sex, race/ethnicity, physical activity level, any vigorous or moderate physical activity, smoking status, and recent health status.

ALT expanded models were adjusted for age, sex, race/ethnicity, physical activity level, alcohol drinks per day, smoking status, and diabetes status.

Abbreviations: ALT, alanine aminotransferase; BMI, body mass index; CI, confidence interval; MEC, Mobile Examination Center; NHANES, National Health and Nutrition Examination Survey; PC, principal component; Estimated VO₂max, estimated maximal oxygen uptake.
