# Supplementary material for: Body-composition phenotype axes provide additional explanatory information for cardiorespiratory fitness and liver-related metabolic risk within BMI strata
Source: Front Nutr. 2026 Aug 31;13:1881228. doi: 10.3389/fnut.2026.1881228 (PMC13572601; doi:10.3389/fnut.2026.1881228)
Supplement: Supplementary file 1 [file Data_Sheet_1.ZIP › Supplementary Table S15.docx]

**Supplementary Table S15.** Primary associations after Benjamini–Hochberg false-discovery-rate correction

Panel A. Multiplicity summary

| Outcome | Primary tests, n | Nominally significant, n | Significant after 24-test BH-FDR, n | Significant after outcome-specific BH-FDR, n |
| --- | --- | --- | --- | --- |
| Estimated VO₂max | 12 | 6 | 5 | 5 |
| ALT | 12 | 8 | 8 | 8 |

Panel B. Estimated VO₂max: β coefficient per 1-SD increment in projected phenotype axis

| BMI quartile | n | Phenotype axis | β coefficient (95% CI) | p value | BH-FDR q value | FDR status |
| --- | --- | --- | --- | --- | --- | --- |
| Q1 | 685 | PC1 | −3.48 (−6.28 to −0.67) | 0.02 | 0.042 | Retained |
| Q1 | 685 | PC2 | 1.46 (0.61 to 2.30) | 0.002 | 0.011 | Retained |
| Q1 | 685 | PC3 | 1.06 (−0.04 to 2.16) | 0.068 | 0.108 | Not retained |
| Q2 | 675 | PC1 | −1.52 (−4.66 to 1.62) | 0.35 | 0.393 | Not retained |
| Q2 | 675 | PC2 | 0.44 (−0.41 to 1.30) | 0.314 | 0.377 | Not retained |
| Q2 | 675 | PC3 | 1.37 (0.16 to 2.59) | 0.033 | 0.057 | Not retained |
| Q3 | 724 | PC1 | −2.81 (−5.83 to 0.20) | 0.076 | 0.114 | Not retained |
| Q3 | 724 | PC2 | 1.82 (0.41 to 3.22) | 0.016 | 0.039 | Retained |
| Q3 | 724 | PC3 | 0.35 (−0.58 to 1.29) | 0.465 | 0.465 | Not retained |
| Q4 | 637 | PC1 | −2.64 (−4.56 to −0.73) | 0.01 | 0.031 | Retained |
| Q4 | 637 | PC2 | 0.58 (−0.15 to 1.30) | 0.127 | 0.179 | Not retained |
| Q4 | 637 | PC3 | 1.72 (0.61 to 2.83) | 0.004 | 0.017 | Retained |

Panel C. ALT: percent difference per 1-SD increment in projected phenotype axis

| BMI quartile | n | Phenotype axis | Percent difference (95% CI) | p value | BH-FDR q value | FDR status |
| --- | --- | --- | --- | --- | --- | --- |
| Q1 | 1102 | PC1 | 19.08% (7.93% to 31.37%) | 0.001 | 0.011 | Retained |
| Q1 | 1102 | PC2 | 1.80% (−2.05% to 5.80%) | 0.37 | 0.393 | Not retained |
| Q1 | 1102 | PC3 | −3.57% (−9.06% to 2.25%) | 0.232 | 0.309 | Not retained |
| Q2 | 1177 | PC1 | 15.62% (2.56% to 30.35%) | 0.023 | 0.043 | Retained |
| Q2 | 1177 | PC2 | 1.67% (−1.94% to 5.41%) | 0.376 | 0.393 | Not retained |
| Q2 | 1177 | PC3 | −2.74% (−7.72% to 2.50%) | 0.306 | 0.377 | Not retained |
| Q3 | 1260 | PC1 | 17.21% (3.05% to 33.31%) | 0.021 | 0.042 | Retained |
| Q3 | 1260 | PC2 | 4.06% (0.91% to 7.31%) | 0.016 | 0.039 | Retained |
| Q3 | 1260 | PC3 | −5.18% (−8.49% to −1.75%) | 0.006 | 0.02 | Retained |
| Q4 | 1318 | PC1 | 17.40% (11.61% to 23.49%) | <0.001 | <0.001 | Retained |
| Q4 | 1318 | PC2 | 6.74% (3.23% to 10.38%) | <0.001 | 0.006 | Retained |
| Q4 | 1318 | PC3 | −6.41% (−10.15% to −2.52%) | 0.003 | 0.014 | Retained |

**Note:**

This table summarizes multiplicity-aware primary associations of projected body-composition phenotype axes with estimated VO₂max and ALT within weighted unified BMI quartiles.

BMI quartiles were defined once from the NHANES analytic base sample using MEC examination weights and applied consistently across outcome analyses.

For estimated VO₂max, effects are adjusted β coefficients with 95% confidence intervals per 1-SD increment in the projected phenotype axis.

For ALT, models were fitted on the natural-log scale and back-transformed; effects are expressed as percent differences in ALT with 95% confidence intervals per 1-SD increment in the projected phenotype axis.

p values are nominal two-sided p values from survey-weighted regression models adjusted for age, sex, and race/ethnicity.

BH-FDR q values were calculated using the Benjamini–Hochberg procedure across all 24 primary stratum-specific tests (3 axes × 4 BMI quartiles × 2 outcomes).

FDR status indicates whether the association remained significant after the 24-test BH-FDR correction using q < 0.05.

Abbreviations: ALT, alanine aminotransferase; BH-FDR, Benjamini–Hochberg false-discovery-rate correction; BMI, body mass index; CI, confidence interval; MEC, Mobile Examination Center; NHANES, National Health and Nutrition Examination Survey; PC, principal component; SD, standard deviation; Estimated VO₂max, estimated maximal oxygen uptake.
