# Supplementary material for: Body-composition phenotype axes provide additional explanatory information for cardiorespiratory fitness and liver-related metabolic risk within BMI strata
Source: Front Nutr. 2026 Aug 31;13:1881228. doi: 10.3389/fnut.2026.1881228 (PMC13572601; doi:10.3389/fnut.2026.1881228)
Supplement: Supplementary file 1 [file Data_Sheet_1.ZIP › Supplementary Table S16.docx]

**Supplementary Table S16.** Formal tests for BMI-stratum × phenotype-axis interactions

| Outcome | Phenotype axis | Analytic n | Design df | Interaction p value | BH-FDR q value | Interpretation |
| --- | --- | --- | --- | --- | --- | --- |
| Estimated VO₂max | PC1 | 2721 | 44 | 0.925 | 0.925 | No FDR evidence of BMI-stratum interaction |
| Estimated VO₂max | PC2 | 2721 | 44 | 0.595 | 0.893 | No FDR evidence of BMI-stratum interaction |
| Estimated VO₂max | PC3 | 2721 | 44 | 0.771 | 0.925 | No FDR evidence of BMI-stratum interaction |
| ALT | PC1 | 4857 | 44 | 0.169 | 0.507 | No FDR evidence of BMI-stratum interaction |
| ALT | PC2 | 4857 | 44 | 0.519 | 0.893 | No FDR evidence of BMI-stratum interaction |
| ALT | PC3 | 4857 | 44 | 0.114 | 0.507 | No FDR evidence of BMI-stratum interaction |

**Note:**

This table reports formal axis-specific tests of interaction between projected phenotype axis and unified BMI quartile in the NHANES external projection sample.

For each outcome and phenotype axis, a survey-weighted regression model included the corresponding projected phenotype axis, unified BMI quartile, their interaction term, age, sex, and race/ethnicity, while accounting for the NHANES multistage survey design.

The interaction p value corresponds to the design-based global test of the phenotype-axis × BMI-quartile interaction. BH-FDR q values were calculated using the Benjamini–Hochberg procedure across the six interaction tests shown in this table.

All q values were >0.05, indicating no false-discovery-rate evidence that the phenotype-axis associations differed statistically across BMI quartiles. Therefore, stratum-specific patterns should be interpreted descriptively rather than as statistically confirmed effect modification.

Abbreviations: BMI, body mass index; BH-FDR, Benjamini–Hochberg false discovery rate; df, degrees of freedom; NHANES, National Health and Nutrition Examination Survey; PC, principal component.
