# Supplementary material for: Body-composition phenotype axes provide additional explanatory information for cardiorespiratory fitness and liver-related metabolic risk within BMI strata
Source: Front Nutr. 2026 Aug 31;13:1881228. doi: 10.3389/fnut.2026.1881228 (PMC13572601; doi:10.3389/fnut.2026.1881228)
Supplement: Supplementary file 1 [file Data_Sheet_1.ZIP › Supplementary Table S17.docx]

**Supplementary Table S17.** Incremental explanatory information provided by projected phenotype axes beyond BMI and demographic covariates

Panel A. Overall model comparison

| Outcome | Scope | n | Design df | Base R² | Extended R² | ΔR² | Wald p | BH-FDR q | Interpretation |
| --- | --- | --- | --- | --- | --- | --- | --- | --- | --- |
| Estimated VO_2_max | Overall | 2721 | 44 | 0.203 | 0.233 | 0.03 | <0.001 | <0.001 | FDR-significant incremental information |
| ALT | Overall | 4857 | 44 | 0.248 | 0.262 | 0.014 | <0.001 | <0.001 | FDR-significant incremental information; small ΔR² |

Panel B. Within-BMI-quartile model comparisons

| Outcome | Scope | n | Design df | Base R² | Extended R² | ΔR² | Wald p | BH-FDR q | Interpretation |
| --- | --- | --- | --- | --- | --- | --- | --- | --- | --- |
| Estimated VO_2_max | Q1 | 685 | 43 | 0.229 | 0.268 | 0.038 | <0.001 | <0.001 | FDR-significant incremental information |
| Estimated VO_2_max | Q2 | 675 | 43 | 0.229 | 0.251 | 0.022 | 0.015 | 0.019 | FDR-significant incremental information; modest ΔR² |
| Estimated VO_2_max | Q3 | 724 | 44 | 0.167 | 0.202 | 0.035 | 0.032 | 0.036 | FDR-significant incremental information |
| Estimated VO_2_max | Q4 | 637 | 44 | 0.148 | 0.2 | 0.051 | <0.001 | <0.001 | FDR-significant incremental information |
| ALT | Q1 | 1102 | 44 | 0.098 | 0.118 | 0.02 | 0.013 | 0.019 | FDR-significant incremental information; modest ΔR² |
| ALT | Q2 | 1177 | 43 | 0.199 | 0.208 | 0.009 | 0.071 | 0.071 | Not FDR-significant |
| ALT | Q3 | 1260 | 44 | 0.194 | 0.209 | 0.015 | <0.001 | <0.001 | FDR-significant incremental information; modest ΔR² |
| ALT | Q4 | 1318 | 44 | 0.267 | 0.298 | 0.032 | <0.001 | <0.001 | FDR-significant incremental information |

**Note:**

Base and extended survey-weighted regression models were compared to quantify the incremental explanatory information provided by the projected phenotype axes. For the overall analysis, the base model included unified BMI quartile, age, sex, and race/ethnicity.

Within each BMI quartile, the base model included age, sex, and race/ethnicity.

The extended model added PC1, PC2, and PC3 to the corresponding base model. R² values are weighted model-based descriptive fit measures; ΔR² was calculated as extended model R² minus base model R² and should be interpreted as explanatory information rather than evidence of clinical prediction, risk reclassification, or treatment-selection utility.

Wald p values correspond to the joint contribution of PC1, PC2, and PC3 in the extended model.

BH-FDR q values were calculated using the Benjamini–Hochberg procedure across the model-comparison tests shown in this table.

Abbreviations: ALT, alanine aminotransferase; BH-FDR, Benjamini–Hochberg false-discovery rate; BMI, body mass index; MEC, Mobile Examination Center; NHANES, National Health and Nutrition Examination Survey; PC, principal component; Estimated VO₂max, estimated maximal oxygen uptake.
