# Supplementary material for: Body-composition phenotype axes provide additional explanatory information for cardiorespiratory fitness and liver-related metabolic risk within BMI strata
Source: Front Nutr. 2026 Aug 31;13:1881228. doi: 10.3389/fnut.2026.1881228 (PMC13572601; doi:10.3389/fnut.2026.1881228)
Supplement: Supplementary file 1 [file Data_Sheet_1.ZIP › Supplementary Table S18.docx]

**Supplementary Table S18.** Pairwise correlations and PCA suitability diagnostics for residualized PCA-ready body-composition inputs

Panel A. Pairwise correlation matrix of residualized PCA-ready body-composition inputs

| Variable | BMI | WHtR | FMI | FFMI | PBF | PhA |
| --- | --- | --- | --- | --- | --- | --- |
| BMI | 1 | 0.966 | 0.958 | 0.876 | 0.867 | 0.436 |
| WHtR | 0.966 | 1 | 0.969 | 0.778 | 0.918 | 0.421 |
| FMI | 0.958 | 0.969 | 1 | 0.725 | 0.951 | 0.331 |
| FFMI | 0.876 | 0.778 | 0.725 | 1 | 0.545 | 0.552 |
| PBF | 0.867 | 0.918 | 0.951 | 0.545 | 1 | 0.287 |
| PhA | 0.436 | 0.421 | 0.331 | 0.552 | 0.287 | 1 |

Panel B. Overall KMO and Bartlett diagnostics

| Diagnostic | Value | Interpretation |
| --- | --- | --- |
| Derivation cohort n used for Bartlett test | 23,698 | Matches the PCA derivation sample |
| Number of PCA-ready variables | 6 | BMI, WHtR, FMI, FFMI, PBF, and PhA |
| Overall KMO | 0.776 | Acceptable sampling adequacy for PCA diagnostics |
| Bartlett test χ² | 288,090.08 | Large departure from an identity correlation matrix |
| Bartlett test df | 15 | Six-variable correlation matrix |
| Bartlett test p value | <0.001 | Supports non-random correlation structure |

Panel C. Variable-level measures of sampling adequacy

| Variable | MSA | Interpretation |
| --- | --- | --- |
| BMI | 0.736 | Acceptable |
| WHtR | 0.95 | Excellent |
| FMI | 0.871 | Good |
| FFMI | 0.633 | Marginal but acceptable |
| PBF | 0.752 | Acceptable |
| PhA | 0.66 | Marginal but acceptable |

**Note:**

Panel A reports pairwise Pearson correlations among the six age- and sex-residualized PCA-ready body-composition inputs in the outpatient derivation cohort. Panel B reports the overall Kaiser–Meyer–Olkin (KMO) measure and Bartlett test of sphericity. Panel C reports variable-level measures of sampling adequacy (MSA).

The Bartlett p value is shown as <0.001 because the computed value underflowed to 0 in the numerical output. These diagnostics support the suitability of PCA as a dimensional-reduction procedure for the residualized input matrix.

However, the high correlations among BMI, WHtR, FMI, and PBF also show substantial structural and mathematical overlap among size and adiposity measures. Therefore, the high cumulative variance explained by the first three components should be interpreted as a compact residualized body-composition covariance structure rather than as evidence that the components represent independent biological dimensions.

Abbreviations: BMI, body mass index; WHtR, waist-to-height ratio; FMI, fat mass index; FFMI, fat-free mass index; PBF, percent body fat; PhA, phase angle; PCA, principal component analysis; KMO, Kaiser–Meyer–Olkin; MSA, measure of sampling adequacy.
