# Supplementary material for: Body-composition phenotype axes provide additional explanatory information for cardiorespiratory fitness and liver-related metabolic risk within BMI strata
Source: Front Nutr. 2026 Aug 31;13:1881228. doi: 10.3389/fnut.2026.1881228 (PMC13572601; doi:10.3389/fnut.2026.1881228)
Supplement: Supplementary file 1 [file Data_Sheet_1.ZIP › Supplementary Table S19.docx]

**Supplementary Table S19**. Effective sample-size and weight-variation diagnostics for NHANES outcome-specific analytic samples

Panel A. Overall outcome-specific analytic samples

| Outcome | Analysis scope | Nominal n | Weighted population total, million | Kish effective n | Approximate design effect from weights | Weight range |
| --- | --- | --- | --- | --- | --- | --- |
| Estimated VO₂max | Overall | 2,721 | 50.8 | 1,996 | 1.36 | 492–69,221 |
| ALT | Overall | 4,857 | 88.02 | 3,452 | 1.41 | 492–69,221 |

Panel B. BMI-quartile-specific analytic samples

| Outcome | Analysis scope | Nominal n | Weighted population total, million | Kish effective n | Approximate design effect from weights | Weight range |
| --- | --- | --- | --- | --- | --- | --- |
| Estimated VO₂max | Q1 | 685 | 13.93 | 536 | 1.28 | 1,908–68,089 |
| Estimated VO₂max | Q2 | 675 | 12.98 | 494 | 1.37 | 1,305–69,221 |
| Estimated VO₂max | Q3 | 724 | 12.98 | 518 | 1.4 | 492–56,428 |
| Estimated VO₂max | Q4 | 637 | 10.91 | 449 | 1.42 | 744–47,187 |
| ALT | Q1 | 1,102 | 21.74 | 838 | 1.32 | 1,042–69,221 |
| ALT | Q2 | 1,177 | 22.1 | 843 | 1.4 | 564–69,221 |
| ALT | Q3 | 1,260 | 22.32 | 879 | 1.43 | 492–56,428 |
| ALT | Q4 | 1,318 | 21.86 | 895 | 1.47 | 544–69,221 |

**Note:**

Kish effective sample size was calculated from the distribution of NHANES examination weights only and is provided as a diagnostic approximation for the outcome-specific analytic samples and BMI-quartile strata. The approximate design effect was calculated as nominal n divided by Kish effective n. These diagnostics do not represent coefficient-specific survey design effects, formal prospective power calculations, or minimum detectable effects. They should be interpreted as indicators of the reduction in effective information due to unequal weighting. BMI quartiles were the weighted unified quartiles used in the primary analyses. Estimated VO₂max denotes the NHANES submaximal treadmill-derived estimate of maximal oxygen uptake.

Abbreviations: ALT, alanine aminotransferase; BMI, body mass index; NHANES, National Health and Nutrition Examination Survey.
