# Supplementary material for: Body-composition phenotype axes provide additional explanatory information for cardiorespiratory fitness and liver-related metabolic risk within BMI strata
Source: Front Nutr. 2026 Aug 31;13:1881228. doi: 10.3389/fnut.2026.1881228 (PMC13572601; doi:10.3389/fnut.2026.1881228)
Supplement: Supplementary file 1 [file Data_Sheet_1.ZIP › Supplementary Table S20.docx]

**Supplementary Table S20.** Exploratory screening of additional NHANES biochemical outcomes in models including projected phenotype axes, with Benjamini–Hochberg adjustment.

**Panel A.** Screened biochemical outcomes and analytic framework

| Outcome | Analytic sample | Weight variable | n | Model scale |
| --- | --- | --- | --- | --- |
| Glucose (GLU) | Fasting subsample | Fasting weight | 2259 | Original scale |
| Insulin (INS) | Fasting subsample | Fasting weight | 717 | Natural-log scale |
| Triglycerides (TG) | Fasting subsample | Fasting weight | 2245 | Natural-log scale |
| Albumin (ALB) | MEC examination sample | MEC weight | 4859 | Original scale |
| Alanine aminotransferase (ALT) | MEC examination sample | MEC weight | 4857 | Natural-log scale |
| Aspartate aminotransferase (AST) | MEC examination sample | MEC weight | 4856 | Natural-log scale |
| HDL cholesterol (HDL) | MEC examination sample | MEC weight | 1609 | Original scale |
| LDL cholesterol (LDL) | MEC examination sample | MEC weight | 2230 | Original scale |
| Serum creatinine (Scr) | MEC examination sample | MEC weight | 4859 | Original scale |
| Total bilirubin (TBIL) | MEC examination sample | MEC weight | 4857 | Natural-log scale |
| Total cholesterol (TC) | MEC examination sample | MEC weight | 4856 | Original scale |
| Total protein (TP) | MEC examination sample | MEC weight | 4855 | Original scale |
| Uric acid (UA) | MEC examination sample | MEC weight | 4857 | Original scale |

**Panel B.** Incremental association screening results with p-values and Benjamini–Hochberg-adjusted q-values.

| Outcome | PC3 effect (95% CI) | PC3 incremental p | PC3 incremental q | Traditional PhA effect (95% CI) | Traditional PhA incremental p | Traditional PhA incremental q | PhA beyond PC1+PC2 p | PhA beyond PC1+PC2 q |
| --- | --- | --- | --- | --- | --- | --- | --- | --- |
| Glucose (GLU), mmol/L | 1.100 (-0.048 to 2.247) | 0.060 | 0.086 | -1.377 (-2.363 to -0.391) | 0.008 | 0.011 | 0.004 | 0.008 |
| Insulin (INS) | -7.40% (-13.53% to -0.84%) | 0.033 | 0.053 | -2.66% (-9.27% to 4.43%) | 0.395 | 0.395 | 0.141 | 0.184 |
| Triglycerides (TG) | -5.68% (-9.88% to -1.28%) | 0.013 | 0.025 | 3.56% (-0.46% to 7.74%) | 0.081 | 0.106 | 0.068 | 0.110 |
| Albumin (ALB), g/L | -0.688 (-0.852 to -0.523) | <0.001 | <0.001 | 0.523 (0.391 to 0.656) | <0.001 | <0.001 | <0.001 | <0.001 |
| Alanine aminotransferase (ALT) | -3.91% (-6.54% to -1.21%) | 0.006 | 0.013 | 5.47% (3.25% to 7.75%) | <0.001 | <0.001 | 0.003 | 0.006 |
| Aspartate aminotransferase (AST) | -0.77% (-2.78% to 1.28%) | 0.448 | 0.448 | 3.50% (1.67% to 5.37%) | <0.001 | <0.001 | 0.552 | 0.552 |
| HDL cholesterol (HDL), mmol/L | 0.023 (-0.013 to 0.058) | 0.172 | 0.186 | -0.013 (-0.044 to 0.018) | 0.359 | 0.389 | 0.399 | 0.432 |
| LDL cholesterol (LDL), mmol/L | -6.093 (-8.281 to -3.905) | <0.001 | <0.001 | 4.979 (3.305 to 6.653) | <0.001 | <0.001 | <0.001 | <0.001 |
| Serum creatinine (Scr), µmol/L | 0.792 (-0.318 to 1.902) | 0.157 | 0.185 | 2.673 (1.599 to 3.746) | <0.001 | <0.001 | 0.114 | 0.164 |
| Total bilirubin (TBIL) | 1.47% (-0.42% to 3.40%) | 0.125 | 0.163 | 1.56% (-0.26% to 3.41%) | 0.090 | 0.107 | 0.338 | 0.400 |
| Total cholesterol (TC), mmol/L | -7.933 (-9.744 to -6.122) | <0.001 | <0.001 | 5.125 (3.476 to 6.773) | <0.001 | <0.001 | <0.001 | <0.001 |
| Total protein (TP), g/L | -1.326 (-1.537 to -1.114) | <0.001 | <0.001 | 0.633 (0.391 to 0.876) | <0.001 | <0.001 | <0.001 | <0.001 |
| Uric acid (UA), µmol/L | -6.668 (-9.952 to -3.383) | <0.001 | <0.001 | 4.318 (1.346 to 7.290) | 0.006 | 0.009 | 0.002 | 0.004 |

**Note:** This exploratory screening extended the main ALT and estimated VO2max analyses to additional NHANES biochemical outcomes. For each outcome, PC3 effect denotes the association of the projected FFMI-dominant phenotype axis with the corresponding biochemical marker; traditional PhA effect denotes the corresponding association for conventional phase angle treated as a single-variable marker. For outcomes modeled on the natural-log scale, effects are presented as percent differences with 95% confidence intervals per 1-SD increment; for outcomes modeled on the original scale, effects are presented as β coefficients with 95% confidence intervals per 1-SD increment. Incremental p-values and q-values summarize the additional contribution of PC3 in the PC1+PC2+PC3 model, the additional contribution of traditional PhA in the conventional model including BMI and WHtR, and the additional contribution of traditional PhA beyond the projected phenotype structure captured by PC1 and PC2. q-values were derived using the Benjamini–Hochberg procedure. These analyses were exploratory and were not part of the prespecified primary evidentiary hierarchy. ALT, alanine aminotransferase; PhA, phase angle.
